# Menin and Menin-Associated Proteins Coregulate Cancer Energy Metabolism

**DOI:** 10.3390/cancers12092715

**Published:** 2020-09-22

**Authors:** Chih-Wei Chou, Xi Tan, Chia-Nung Hung, Brandon Lieberman, Meizhen Chen, Meena Kusi, Kohzoh Mitsuya, Chun-Lin Lin, Masahiro Morita, Zhijie Liu, Chun-Liang Chen, Tim Hui-Ming Huang

**Affiliations:** 1 Department of Molecular Medicine, University of Texas Health Science Center at San Antonio, San Antonio, TX 78229, USA; ChouC@uthscsa.edu (C.-W.C.); TanX3@uthscsa.edu (X.T.); HungC@uthscsa.edu (C.-N.H.); lieb808@hawaii.edu (B.L.); CHENM1@uthscsa.edu (M.C.); KusiM@livemail.uthscsa.edu (M.K.); Mitsuya@uthscsa.edu (K.M.); chunlin.lin2014@gmail.com (C.-L.L.); MoritaM@uthscsa.edu (M.M.); LiuZ7@uthscsa.edu (Z.L.); 2 Department of Life Science, Tunghai University, Taichung 407, Taiwan

**Keywords:** menin, menin-associated proteins, oxidative phosphorylation, glycolysis, circulating tumor cells

## Abstract

**Simple Summary:**

Maintaining the energy stability is critical for cell surviving and adapting in a vagary environment. Glycolysis and mitochondrial oxidative phosphorylation (OXPHOS) are two major energy production pathways in cells. Here we demonstrated that menin regulates the expression of OXPHOS and glycolytic genes, and this regulation can further be modified by a group of menin-associated proteins (MAPs) including KMT2A, MED12, WAPL, and GATA3. Downregulation of menin and MAP genes altered the proportion of glycolysis and OXPHOS for energy generation, and we found a counteracting function of menin and MAPs when the shRNA knockdown cells are exposed to metabolic stress. Menin and MAPs may serve as transcriptional sensors for balancing the preference between glycolysis and OXPHOS. This coordinated regulation is crucial for cell adaption to stressful microenvironments using different pathways for energy production.

**Abstract:**

The interplay between glycolysis and mitochondrial oxidative phosphorylation (OXPHOS) is central to maintain energy homeostasis. It remains to be determined whether there is a mechanism governing metabolic fluxes based on substrate availability in microenvironments. Here we show that menin is a key transcription factor regulating the expression of OXPHOS and glycolytic genes in cancer cells and primary tumors with poor prognosis. A group of menin-associated proteins (MAPs), including KMT2A, MED12, WAPL, and GATA3, is found to restrain menin’s full function in this transcription regulation. shRNA knockdowns of menin and MAPs result in reduced ATP production with proportional alterations of cellular energy generated through glycolysis and OXPHOS. When shRNA knockdown cells are exposed to metabolic stress, the dual functionality can clearly be distinguished among these metabolic regulators. A MAP can negatively counteract the regulatory mode of menin for OXPHOS while the same protein positively influences glycolysis. A close-proximity interaction between menin and MAPs allows transcriptional regulation for metabolic adjustment. This coordinate regulation by menin and MAPs is necessary for cells to rapidly adapt to fluctuating microenvironments and to maintain essential metabolic functions.

## 1. Introduction

Menin encoded by the *MEN1* gene is a multifaceted protein participating in a variety of cellular functions [1]. Earlier studies revealed that germline mutations accompanied by somatic alterations of *MEN1* lead to the development of multiple endocrine neoplasia type 1 in endocrine glands [2]. Through a two-hit mechanism, this tumor-suppressor inactivation has also been observed in lung, breast, and gastrointestinal cancers [3,4,5]. Although *MEN1* is well recognized as a classical tumor-suppressor gene, subsequent studies found aberrant upregulation of the gene in solid tumors and leukemias [6,7]. The gene product, menin, appears to interact with oncoproteins to promote transcription programs for cellular transformation and proliferation [8,9]. In particular, menin forms a complex with KMT2A (formerly MLL1) or other family proteins to activate gene transcription via histone methylation and deacetylation in solid tumors [10,11]. Recent findings indicated that menin collaborates with hormone signaling involved in the progression of breast and prostate cancers [10,12]. Indeed, the initial evidence points to complex regulatory roles of menin in response to different physiological conditions [13].

A growing body of studies has now implicated that menin acts as a transcriptional driver, engaging in two regulatory events for tumor-promoting and tumor-suppressing activities, respectively [13,14]. To do so, menin must interact with different factors to specify the active or suppressive mode for regulating gene transcription. Biochemical and crystal structural analyses revealed that menin is a scaffold protein providing multiple docking sites for transcriptional machinery components [1,14,15]. Further studies found that menin individually cooperates with oncogenic signaling components, including TGF-β/SMDAs, NFκB, β-catenin, estrogen receptor alpha (ERα), and an androgen receptor, for transcriptional modulation of downstream targets responsible for cellular transformation and proliferation, DNA replication and repair, chromatin modification, and hormone resistance [9,12,16,17,18]. While these studies give insights into a multifaceted role of menin in oncogenesis, it is still a challenge to know how menin interacts with diverse protein partners in cancer cells responding to different microenvironments.

To this end, we combined RNA-seq transcriptomic profiles, BioID protein–protein interactions [19], and in silico correlation comparisons to investigate crucial functions of menin in breast cancer cells. The rationale was that menin had been implicated as a proliferative determinant during breast tumorigenesis [10]. In addition to confirming a role of menin in ERα-modulated signaling [10,18], our integrated analysis identified a group of menin-associated proteins (MAPs) for transcriptional regulation of genes involved in metabolic energy pathways, including glycolysis and mitochondrial oxidative phosphorylation (OXPHOS) [20]. As a positive modulator, menin interacts with a MAP for metabolic regulation in a context-dependent manner. Whereas a MAP negatively influences OXPHOS, the same protein may positively regulate glycolysis. Furthermore, we set out to demonstrate that close-proximity interactions between these proteins are essential for the coregulation of metabolic gene transcription in response to microenvironmental changes.

## 2. Results

### 2.1. Gene Transcription of Energy Metabolism Pathways Modulated by MEN1

**Figure 1:**
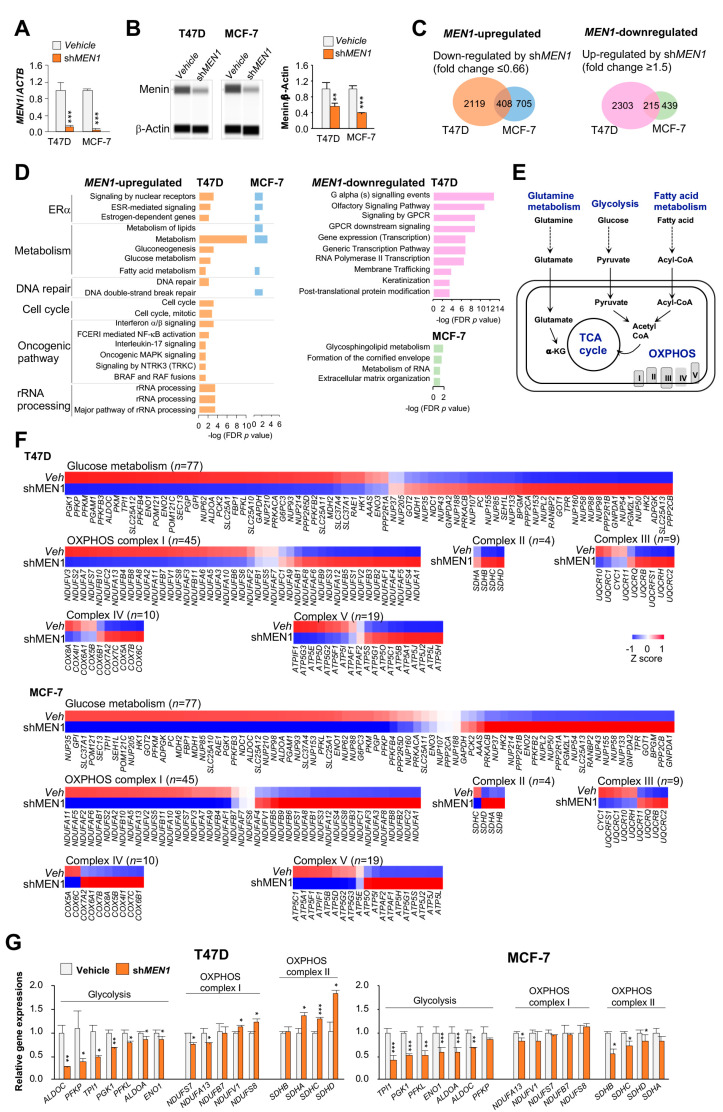
Identification of *MEN1-*modulated genes in breast cancer cells. (**A**) RT-qPCR of *MEN1* in T47D or MCF-7 cells treated with vehicle or *MEN1* shRNA lentivirus (*n* = 3). (**B**) Quantitative Western immunoassays (WES) of menin expression in T47D or MCF-7 cells treated with vehicle or *MEN1* shRNA lentivirus (*n* = 3). (**C**) Venn diagrams of differentially expressed genes (fold change ≥1.5 or ≤0.66) in T47D or MCF-7 cells after sh*MEN1* knockdown compared with vehicle controls (*n* = 2). (**D**) Pathway annotation analysis of *MEN1*-upregulated and *MEN1*-downregulated genes in T47D or MCF-7 cells using DAVID including cancer hallmark pathways. (**E**) Schematic illustration of five major metabolic pathways. (**F**) Expression heat maps of oxidative phosphorylation (OXPHOS) and glycolytic genes in both *MEN1* knockdown T47D and MCF-7 cells (fold changes relative to vehicle controls). (**G**) Bar charts of the expression levels of representative OXPHOS and glycolytic genes affected by *MEN1* knockdown in T47D or MCF-7 cells using RT-qPCR. Data are presented as mean ± S.D. Unpaired two-tailed Student’s *t*-test was used for statistics. * *p* < 0.05, ** *p* < 0.01, and *** *p* < 0.001.

Increased *MEN1* expression was found in primary breast tumors relative to normal controls in The Cancer Genome Atlas (TCGA) cohort (*p* < 0.001, Figure S1A). The upregulation was positively associated with elevated *MEN1* copy-numbers (*p* < 0.0001, Figure S1B). In addition, patients with ERα-positive tumors that displayed high expression levels or elevated copy-numbers of *MEN1* appeared to have poor prognosis (Figure S1C,D). To further investigate how this aberrant expression contributes to tumorigenesis, we performed an RNA-seq analysis of *MEN1* shRNA knockdown in two ERα-positive breast cancer cell lines T47D and MCF-7, respectively (Figure 1A,B). Differentially expressed genes (i.e., fold change ≥1.5 and ≤0.66 for upregulated and downregulated genes, respectively, from two biological repeats) were identified in both shRNA knockdown cells relative to vehicle controls. Although the knockdown efficiencies (i.e., 88–95% at the RNA level and 40–60% at the protein level) were comparable in both cell lines, more differentially expressed genes were observed in T47D knockdown cells than in MCF-7 knockdown cells (Figure 1C). The gene set enrichment analysis also reflected more notable impacts of menin on signaling transduction pathways in T47D cells relative to MCF-7 cells (Figure 1D). Consistent with a previous finding [10], the pathway analysis confirmed a role of menin in regulating ERα signaling in breast cancer cells. Surprisingly, we found that metabolic energy pathways were the most affected by *MEN1* knockdown (Figure 1D,E). In particular, the *MEN1* knockdown had a greater influence on genes encoding structural components and regulatory functions for glycolysis and OXPHOS (Figure 1F). The RT-qPCR analysis of 16 representative genes involved in glycolysis and in two mitochondrial electron transport chains (i.e., OXPHOS complexes I and II) further confirmed the robustness of RNA-seq data (Figure 1G). Collectively, the results find a previously unknown role of menin in regulating metabolic energy pathways.

### 2.2. Energy Metabolism Genes Coregulated by Menin and Menin-Associated Proteins in Primary Tumors

**Figure 2:**
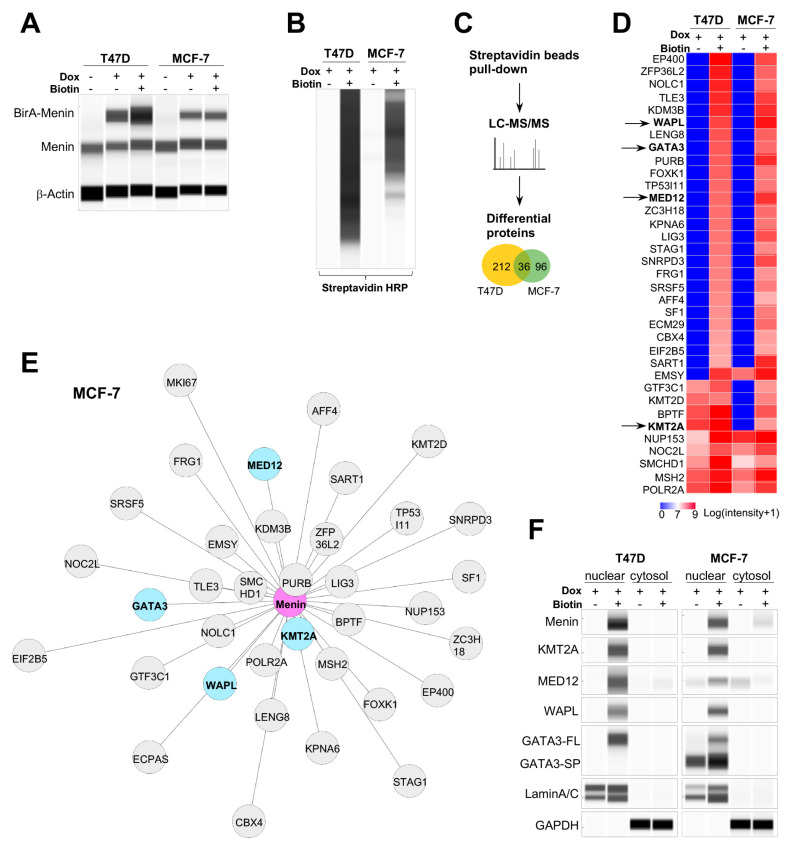
Identification of menin-associated proteins (MAPs) in breast cancer cells. (**A**) and (**B**) WES of BirA-Menin fusion proteins (**A**) and biotin-labeled proteins (**B**) in total lysates of *BirA-MEN1* BioID engineered T47D or MCF-7 cells after incubating with or without doxycycline and biotin. (**C**) Schematic purification and proteomic identification of MAPs using LC–MS/MS. (**D**) Heatmap of the quantification of 35 MAPs commonly shared in T47D and MCF-7 cells. MAPs further verified by WES immunoassays were indicated by arrows. (**E**) Network analysis of 35 MAPs in MCF-7 cells. The distance between menin and MAPs represented the quantitative ratio of each MAP and menin. MAPs marked in blue were further assayed by WES. (**F**) Nuclear or cytoplasmic lysates of *BirA-MEN1* BioID engineered T47D or MCF-7 cells after streptavidin beads pull-down were detected by WES with antibodies against menin, KMT2A, MED12, WAPL, GATA3, LaminA/C, or GAPDH. FL, full length; SP, spliced form.

As menin is a putative metabolic regulator, we postulated that this protein acts as a crucial component of a transcription complex to bind or interact with other factors for gene regulation. Therefore, we used a proximity-based biotin labeling technique or BioID to identify menin-associated proteins (MAPs) in T47D and MCF-7 cells (Figure S2A; details in Methods). Proteins within a 10 nm radial proximity of ligase-fused menin were biotinylated in breast cancer cells transduced with doxycycline-inducible BirA-menin in the presence of biotin and doxycycline, and the labeled proteins were pulled down by streptavidin-beads (Figure 2A,B) [19,21]. Analysis of liquid chromatography–mass spectroscopy (LC–MS/MS) on biotinylated proteins identified 248 and 132 putative nuclear MAPs in T47D and MCF-7 cells, respectively (Figure 2C). Based on known functions, MAPs were categorized into eight groups involved in basal transcription, DNA repair, mRNA modeling, nuclear scaffolding, site-specific transcription, cohesin complexes, and histone modification (Table S1). Of the 35 MAPs shared by T47D and MCF-7 cells, ten were detectable even in control cells without biotin added, suggesting the presence of low endogenous biotin and probably their strong interactions with menin (Figure 2D). The spatial nearness of individual MAPs to menin was estimated according to biotinylated protein quantities (Figure 2E; Figure S2B). To check the robustness of BioID findings, capillary Western immunoassays (WES) were conducted on nuclear and cytoplasmic lysates of T47D and MCF-7 cells, respectively, using specific antibodies against menin and four representative MAPs—KMT2A, MED12, WAPL, and GATA3 (Figure 2F). The assays confirmed the association of these MAPs with menin was predominant in the nuclei, implicating their partnership with menin in gene regulation.

We further performed an in silico analysis of chromatin immunoprecipitation sequencing (ChIP-seq) data using anti-menin and anti-KMT2A [10]. The menin and KMT2A binding sites are localized in the proximity of promoters and gene bodies of representative glycolysis and OXPHOS genes, suggesting a transcription regulatory role of menin and KMTs on these genes (Figure S2C,D). Some of the menin and KMT2A binding sites are colocalized in some regions of gene loci. These confirm that menin and KMT2A are closely associated and likely coregulate these energy metabolism genes.

**Figure 3:**
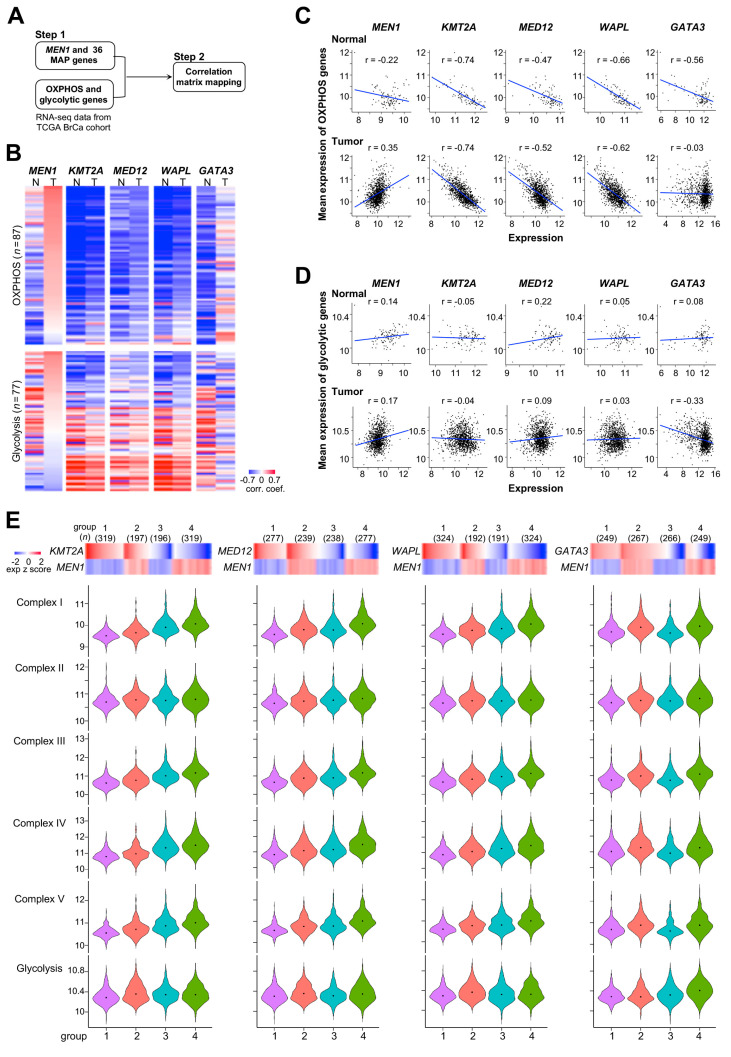
Expression correlation relationship of menin/MAPs genes and OXPHOS/glycolytic genes. (**A**) Workflow of the in silico correlation analysis of gene expression in The Cancer Genome Atlas (TCGA) breast cancer cohort. (**B**) Heatmaps of the expression correlation between *MEN1*/selected 4 MAP genes and OXPHOS genes (upper) or glycolytic genes (lower) in normal (N) and tumor (T) samples. The genes are arranged from the highest to the lowest according to gene expression correlation coefficients of *MEN1*–OXPHOS genes or *MEN1*–glycolytic genes in breast tumors. (**C**) and (**D**) Scatter plots and linear regression analyses of MEN1/selected MAPs expression and mean expression of OXPHOS genes (**C**) or glycolytic genes (**D**) in normal and tumor samples. (**E**) Violin plots (lower panel) shows the average expressions of the genes of OXPHOS complexes I-V and glycolysis in the samples of each of the corresponding 4 groups are shown as violin plots. Based on the median values (where ≥median is “high” and <median is “low”) of the expression of the corresponding individual genes (*KMT2A*, *MEN1*2, *WAPL*, and *GATA3*) and *MEN1*, the TCGA breast tumor samples were divided into 4 groups—1: high-low, 2: high-high, 3: low-low, and 4: low-high (upper panel). Letters on top of the violin plot denote statistical significance, where two groups with different letters are significantly different (*p* < 0.05) and those with the same letter are not.

Since the role of MAPs in controlling energy metabolism remains to be explored, we sought to determine the coexpression relationship between menin/MAP genes and OXPHOS/glycolytic genes. In silico analysis generated pairwise expression correlation heatmaps in 1031 primary breast tumors and 98 normal controls of the TCGA cohort (Figure 3A,B; Figure S3A,B). We observed that expression of *MEN1* was moderately negatively correlated (*r* = −0.22) with the mean OXPHOS gene expression in normal controls but both turned to being positively correlated (*r* = 0.35) as they progressed into malignant tumors (Figure 3C). In contrast, increased expression of three MAP genes—*KMT2A*, *MED12*, and *WAPL—*showed strong negative correlation with OXPHOS expression in both tumors and controls while *GATA3* exhibited no correlation. It is notable that the expression of *MEN1* was also positively correlated with that of glycolytic genes, although to a lesser degree than OXPHOS genes (Figure 3D). There was no expression correlation between glycolytic genes and *KMT2A*, *MED12*, or *WAPL*, except for *GATA3* showing a weak negative association. Next, we determined the coexpression relationship between MAP genes and *MEN1* in primary tumors that were divided into four groups based on median expression levels: 1: high (above the median value) MAP and low (below the median value) *MEN1*; 2: high MAP and high *MEN1*; 3: low MAP and low *MEN1*; and 4: low MAP and high *MEN1* (Figure 3E and Figure S3C). A trend of increased mean expression of OXPHOS genes (complex I-V) could be observed from Group 1 to 4. For example, high *KMT2A* and low *MEN1* expressed tumors (Group 1) displayed lowest OXPHOS expression. In contrast, tumors with a reverse expression pattern of *KMT2A* and *MEN1* (Group 4) had the highest OXPHOS expression (Figure 3—left panel). Based on the data here and the forementioned menin1 as a positive regulator for OXPHOS gene expression, *KMT2A* seems to suppress *MEN1* function. This negative regulation was also seen between *MEN1* and two other MAP genes (*MED12* and *WAPL*, Figure 3E—middle two panels), but was not evident for *GATA3*. However, the negative coexpression relationship of *MEN1* and three MAPs was not observed for glycolytic gene expression in the Group 1–4 tumors. Overall, the in silico analysis suggests an essential role of menin in upregulating cancer energy metabolism and the three MAPs may counteract menin, particularly in the transcription of OXPHOS genes.

### 2.3. Counteracting Roles of MAPs in Menin-Modulated OXPHOS and Glycolysis

**Figure 4:**
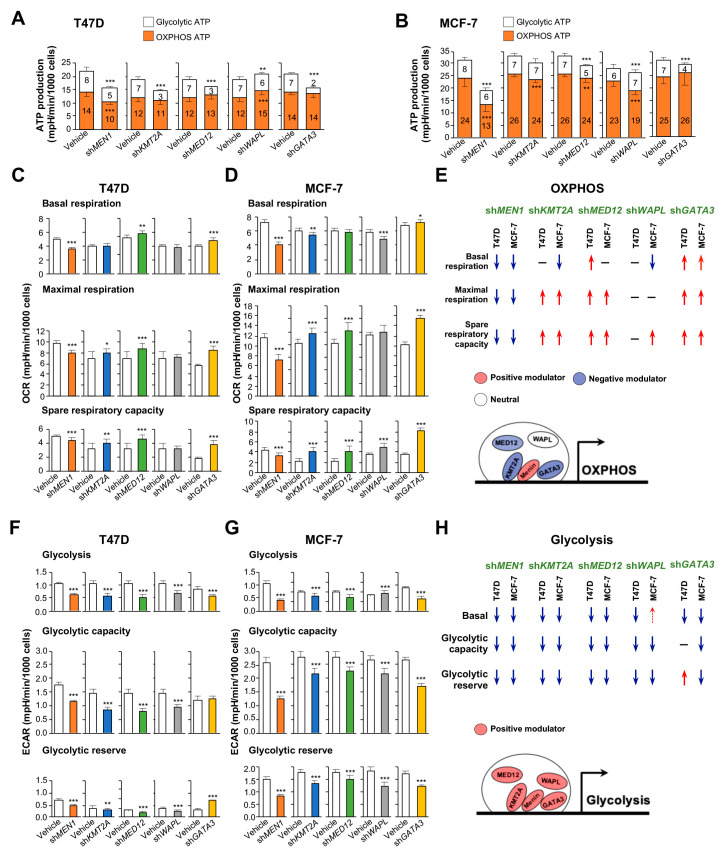
Bioenergetic dynamics are regulated by menin and MAPs in T47D and MCF-7 cells. (**A**) and (**B**) Glycolytic and OXPHOS ATP productions in T47D (**A**) or MCF-7 (**B**) cells infected with vehicle, sh*MEN1*, sh*KMT2A*, sh*MED12*, sh*WAPL*, or sh*GATA3* lentivirus. Statistics represented the difference of glycolytic or OXPHOS ATP production between shRNA knockdown and vehicle controls. (**C**) and (**D**) Bar charts representing mitochondrial functions in the single knockdown of *MEN1*, *KMT2A*, *MED12*, *WAPL*, or *GATA3* and their vehicle control in T47D (**C**) or MCF-7 (**D**) cells. (**E**) Schematic summary of mitochondrial dynamics affected by the knockdown of *MEN1* or MAPs. (**F**,**G**) Bar charts represented the glycolytic functions in T47D (**F**) or MCF-7 (**G**) cells subject to gene knockdown by sh*MEN1*, sh*KMT2A*, sh*MED12*, sh*WAPL*, or sh*GATA3* lentivirus. (**H**) Schematic summary of glycolytic functions affected by the knockdown of *MEN1* or MAPs. Data are presented as mean ± S.D. (*n* = 15–20 technical-replicate wells). Statistical significance was performed by an unpaired two-tailed Student’s *t*-test between treated groups and corresponding controls. * *p* < 0.05, ** *p* < 0.01, and *** *p* < 0.001.

To mechanistically validate menin/MAPs-coregulated energy metabolism outcomes, we first carried out shRNA knockdowns of *MEN1* and four MAP genes (*KMT2A*, *MED12*, *WAPL*, and *GATA3*), which led to expression alterations of glycolytic and OXPHOS genes analyzed in T47D and MCF-7 cells (see examples in Figure 1G and Figure S4A–C). Then, we determined whether these knockdowns affect ATP profiles in breast cancer cells based on the oxygen consumption rate (OCR) and extracellular acidification rate (ECAR) assays. The *MEN1* knockdown resulted in reduced ATP production from both OXPHOS and glycolysis in T47D and MCF-7 cells (Figure 4A,B). Individual knockdowns for *KMT2A*, *MED12*, *WAPL*, or *GATA3* suppressed the overall ATP generation to a lesser degree than the *MEN1* knockdown (Figure 4A,B). The finding demonstrates that MAPs appeared to be regulators of ATP synthesis derived from the two metabolic pathways.

To explore how menin or a MAP affects different OXPHOS processes, we added inhibitors to induce metabolic stress in shRNA knockdown cells or vehicle controls (Figure S4D–F). Prior to the inhibition, *MEN1* knockdown caused low basal OCRs, consistent with attenuated ATP production from OXPHOS in T47D and MCF-7 cells (*p* < 0.001, Figure 4C,D—upper panels). Basal respiration showed a slight increase or decrease in *KMT2A*, *MED12*, *WAPL*, or *GATA3* knockdown cells compared to vehicle controls. When the mitochondrial membrane potential was additionally disrupted by carbonyl cyanide-4 (trifluoromethoxy) phenylhydrazone (FCCP) [22], *MEN1* knockdown cells exhibited defects in both maximal respiration potential and spare respiratory capacity for energy demand (Figure 4C,D—middle and lower panels). The findings indicated that menin plays a crucial role in the modulation of these OXPHOS functions. On the contrary, when *KMT2A*, *MED12*, or *GATA3* was knocked down, we observed an increase in both maximal and spare respiration rates in these cells, suggesting that these four MAPs are negative modulators of OXPHOS activities that are mainly consistent with transcription reduction (Figure 3C,E and Figure 4E).

We also investigated the glycolytic functions of cells subjected to shRNA knockdown and metabolic stress (i.e., transient glucose depletion for 1 h; Figure S4G–I). When glucose was added back to the media, all five knockdown cells (*MEN1*, *KMT2A*, *MED12, WAPL*, and *GATA3*) displayed an attenuated ability to fully utilize glucose via glycolysis (Figure 4F,G—upper panels). In addition, there was a general decrease of utilizing the glycolytic reserve in knockdown cells, except for T47D cells with *GATA3* knockdown (Figure 4F,G—middle and lower panels). Taken together, these metabolic stress tests found that menin and the four MAPs mainly act as positive regulators of glycolysis although primary tumor transcription data do not show the relevance (Figure 3D,E and Figure 4H).

### 2.4. Essential Interactions between Menin and KMT2A for OXPHOS Functions

**Figure 5:**
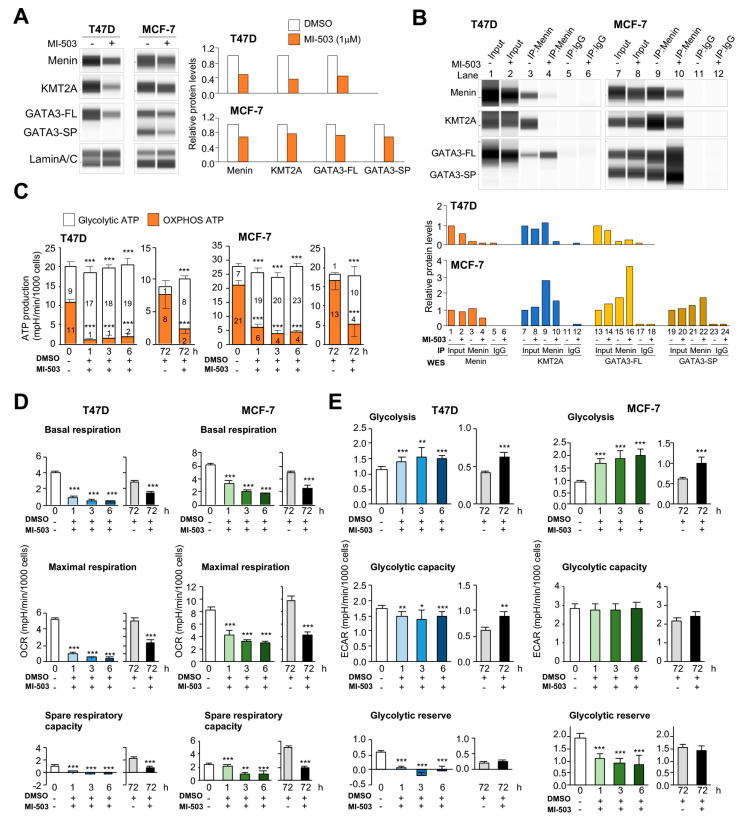
Integrity of the menin–KMT2A complex is required for OXPHOS functions. (**A**) WES of T47D or MCF-7 cells treated with DMSO or 1 μM MI-503 for 3 days (left). Relative protein expression normalized to the average of LaminA/C in WES (right). FL, full length; SP, spliced form. (**B**) Nuclear lysates of T47D or MCF-7 cells treated with DMSO or 1 μM of MI-503 for 3 days were immunoprecipitated with the menin antibody or IgG, and assayed by WES (upper). Relative protein expression in WES (lower). The protein expression in DMSO treated input was normalized as 1. FL, full length; SP, spliced form. (**C**) Glycolytic or OXPHOS ATP production in T47D or MCF-7 cells treated with 1 μM MI-503 for 0, 1, 3, 6, and 72 h, or DMSO control for 72 h. (**D**,**E**) Bar charts of the Seahorse mitochondrial stress test (**D**) and glycolytic stress test (**E**) on T47D or MCF-7 cells treated with 1 μM MI-503 for 0, 1, 3, 6, and 72 h, or DMSO for 72 h. Data are presented as mean ± S.D. (*n* = 10–15 technical-replicate wells). An unpaired two-tailed Student’s *t*-test was used to determine statistical significance for the difference between MI-503-treated groups and its controls. * *p* < 0.05, ** *p* < 0.01, and *** *p* < 0.001.

The above findings prompted us to investigate whether physical interactions between menin and MAPs are required for metabolic regulation. To this end, we determined how the small molecule inhibitor MI-503 [23] disrupts the menin-KMT2A interaction that negatively influences downstream ATP production in breast cancer cells. After optimizing treatment doses based on a cell viability test (Figure S5A), we treated T47D and MCF-7 cells with or without 1 μM MI-503 for 72 h and then determined the status of menin–MAP interactions using coimmunoprecipitation and WES. MI-503 treatment caused a modest loss of menin and two MAP proteins—KMT2A and GATA3—likely resulting in an unstable menin complex that may contribute to a decline in cell viability (Figure 5A). The presence of MI-503 greatly abolished the menin and KMT2A interaction but led to a 2-fold increase in the binding between menin and a GATA3 variant (GATA3-FL) in T47D cells (Figure 5B). The menin-KMT2A interaction was also affected in MI-503-treated MCF-7 cells to a lesser degree (0.5×; Figure 5B). However, we observed a 1.5-fold increase in the binding between menin and two GATA3 variants (GATA3-FL and GATA3-SP) in MCF-7 cells. In addition to KMT2A and GATA3, we also demonstrated that another MAP, WAPL, also coimmunoprecipitated with menin in T47D providing another line evidence of physical association between menin and MAPs (Figure S5B).

Although the possibility of MI-503’s direct blocking of OXPHOS ATP production was not ruled out, cells at 1–6 h post-treatment by MI-503 showed a transient decrease in OXPHOS gene expression, but could partially be recovered to the original levels at 72 h (Figure S5C), indicating a transcriptional regulation by menin-MAPs. As a result, a drastic shift of ATP production from OXPHOS to glycolysis occurred after treating breast cancer cells with MI-503 (Figure 5C). The reduced ATP synthesis from OXPHOS was largely reflective of diminished basal, maximal, and spare respiration capacity at 1–6 h after the treatment (Figure 5D and Figure S5E). This respiratory inhibition was slightly reversed at 72 h post-treatment. We postulated that the attenuated OXPHOS transcription could be attributed in part to the disruption of menin-KMT2A by MI-503, leading to the defects in OXPHOS machineries.

Dissociation of menin-KMT2A, however, did not reduce glycolytic ATP generation in MI-503-treated cells while there were temporal expression changes of glycolytic genes (Figure S5D). Instead, we observed a sharp increase in the glycolytic ATP level to compensate for the loss of OXPHOS ATPs in treated cells. Metabolic fluxes might contribute to this energy compensation [24], even though the expression of the corresponding genes was transiently affected by MI-503. When glucose was added back to the media after a 1 h depletion in the metabolic stress assay, MI-503-treated cells displayed an even greater ability than untreated cells to utilize glycolysis (Figure 5E and Figure S5F). While the level of glycolytic capacity remained stable at 1–6 h after the treatment, the glycolytic reserve became largely depleted in treated cells. In this case, ATP compensation came predominantly from the glycolytic reserve function when the OXPHOS pathway was blocked in MI-503-treated cells. At 72 h, we observed that the overall glycolytic function could be even more efficiently restored than OXPHOS. While dimethyl sulfoxide (DMSO), the solvent to dissolve MI-503, is known to negatively affect cellular glucose uptake [25], these glycolytic functions seemed to work better in MI-503-treated cells than in control cells exposed to long-term DMSO. The finding might be compatible with an overall recovery of glycolytic gene expression in cancer cells after a long-term 72 h treatment with MI-503. Although the menin-KMT2A disruption negatively influences glycolytic gene expression, our finding suggests that this transient interference may not have an adverse effect on downstream glycolysis function, which is used to compensate for ATP loss from OXPHOS.

### 2.5. Upregulation of MEN1, OXPHOS, and Glycolytic Genes in Circulating Tumor Cells

**Figure 6:**
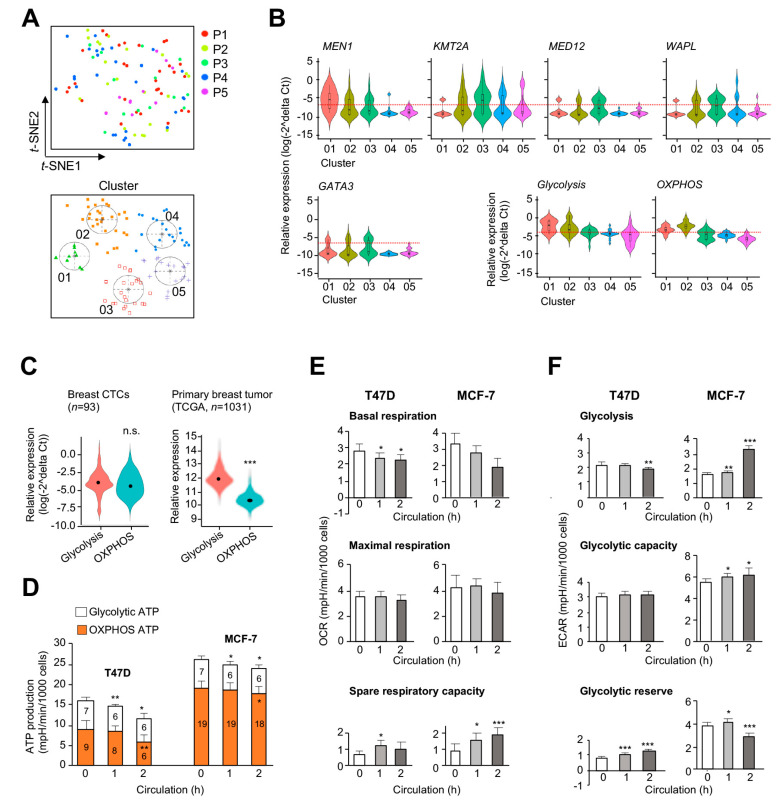
*MEN1* and OXPHOS expression are increased in breast circulating tumor cells (CTCs). (**A**) t-SNE profile plots and cell clustering of 93 CTCs from 5 breast cancer patients based on the single cell RT-qPCR expression profiling of 11 OXPHOS genes (*NDUFA7*, *NDUFA11*, *NDUFA13*, *NDUFB7*, *NDUFS7*, *NDUFS8*, *NDUFV1*, *SDHA*, *SDHB*, *SDHC*, and *SDHD*). (**B**) Violin plots of *MEN1* or selected MAPs expression, mean expression of 7 glycolytic genes (*ALDOA*, *ALDOC*, *ENO1*, *PFKL*, *PFKP*, *PGK1*, and *TPI1*) or mean expression of 11 OXPHOS genes (aforementioned) in the five cell clusters. Statistical significance among clusters was carried out using the Duncan multi-range test. (**C**) Mean expression of 7 glycolytic genes and 11 OXPHOS genes in these 93 breast CTCs or in the TCGA primary breast cancer cohort. (**D**) Glycolytic and OXPHOS ATP productions of T47D or MCF-7 cells after circulation (*n* = 6–10 technical replicates). (**E**,**F**) Mitochondrial (**E**) and glycolytic (**F**) functions of T47D or MCF-7 cells after circulation (*n* = 5–9 technical-replicate wells). Statistics represented the difference between no circulating control and each treatment. Data are presented as mean ± S.D. An unpaired two-tailed Student’s *t*-test was used for statistical significance determination. * *p* < 0.05, ** *p* < 0.01, and *** *p* < 0.001.

We next used circulating tumor cells (CTCs) as in vivo surrogates to validate our findings based on cultured cell models and in silico analyses (Figure S6A). CTCs isolated from five breast cancer patients were briefly cultured in a sphere-forming medium (#91130, Fujifilm Irvine Scientific, Santa Clara, CA, USA) for enrichment [26]. Epithelial-like and cancer-stemness features of CTCs were confirmed with an immunofluorescence analysis [27,28] (Figure S6B). Single-cell RT-qPCR of 93 CTCs was conducted with a gene panel of *MEN1*, *KMT2A*, *MED12, WAPL*, and *GATA3* and 18 representative genes of OXPHOS and glycolysis. t-distributed stochastic neighbor embedding (*t*-SNE) profiling identified five clusters (#01–05; Figure 6A), and violin plots displayed CTC clusters from high to low expression levels of *MEN1* (Figure 6B). Cluster #01 and 02 had high *MEN1* expression, which also corresponded to increased levels of OXPHOS and glycolytic genes (Figure 6B). Besides, the expression levels of the four MAP genes—*KMT2A*, *MED12, WAPL*, and *GATA3*—were lower than that of *MEN1* in these two clusters.

The high expression profiles of OXPHOS and glycolytic genes in CTCs (particularly those in #01 and 02) were distinct from those observed in TCGA primary tumors, known to preferentially depend on glycolysis for the Warburg metabolism [29] (Figure 6C). Therefore, we suggest that high OXPHOS and glycolysis activities can be a metabolic adaptation of CTCs to survive under biophysical and physiological stress during the blood circulation [30]. To further investigate the impact of this microenvironmental change on energy metabolism, T47D and MCF-7 cells were subjected to circulation in an in vitro system simulating capillary flows [31] (Figure S6C). After 2 h in the circulation, less than 50% of cells were viable for ATP measurements (Figure S6D). These post-circulation cancer cells displayed slight decreases of ATP production from both glycolysis and OXPHOS (Figure 6D). When these cells were subjected to metabolic stress tests, basal respiration rates were slightly lowered in post-circulation cells, compared to non-circulation controls (Figure 6E—upper panels). However, maximal rates remained unchanged due to the contribution of spare respiratory capacity from these post-circulation cells (Figure 6E). Although T47D and MCF-7 cells responded differently with respect to their glycolytic functions, the glycolytic reserve was moderately increased in both cell lines (Figure 6F). Taken together, we suggest that the enhancement of both spare/reserve energy capacities could be partially responsible for the survival of cancer cells in the blood circulation.

## 3. Discussion

Glycolysis and OXPHOS are two major metabolic pathways to provide sufficient ATP for the maintenance of essential cellular functions and cancer growth [20]. These pathways are tightly regulated, depending on the availability of oxygen or glucose in microenvironments [32]. While previous reports focused on post-translational and post-transcriptional controls of glycolysis and OXHPOS, emerging evidence reveals that metabolic genes are frequently coregulated at the transcriptional level [33,34,35,32]. A few transcription factors are known to have a direct role in the regulation of energy metabolism [34,37,38]. For example, HIF-1α or SIX1 binds to conserved DNA motifs in promoter regions of multiple glycolytic genes, enhancing the transcription for the biosynthesis of glycolytic pathway enzymes [32,39]. Genomic analysis also revealed coregulatory modules for synchronized transcription of OXPHOS genes [33,35]. Menin promotes glycolysis through MYC for tumor progression [8]. Our present findings further implicate that menin acts as a key factor to concordantly regulate the transcription machineries of both metabolic pathways. The coordinate regulation by menin is necessary for cells to constantly maintain energy homeostasis and to rapidly adapt to fluctuating microenvironments.

Assisting in menin-mediated transcription is a group of coregulatory partners or MAPs based on a protein–protein interaction assay based on the data from the four representative MAPs as more relation details remain to be determined. With limited data of the shRNA knockdown effect on OCR and ECAR, WAPL, GATA3, and MED12 may act like KMT2A as menin’s coregulators in energy metabolism as depicted in Figure 4E,H. These four and likely other MAPs may compete for association or binding with menin as more GATA3 isoforms are coimmunoprecipitated with menin when KMT2A-menin binding is interfered by MI-503. The menin–MAPs complex dynamics may be regulated by menin upstream signaling pathways and also important for the regulation of energy metabolism.

While menin is a positive regulator of glycolysis and OXPHOS, MAPs contribute to transcriptional switches between the two pathways. For example, KMT2A may counteract the positive regulatory mode of menin for OXPHOS transcription although the same protein facilitates active transcription of many glycolytic genes. It has been suggested that KMT2A interacts with multiple partners and is cleaved by taspase 1 into two fragments inside a cell—the N-terminal domain, known to bind transcription repressors, and the C-terminal domain, which is a transcriptional activator [40,41]. Future investigation is warranted to determine if the two protein substructures independently regulate the transcription of OXPHOS and glycolytic genes. Nevertheless, this transcriptional coregulation is needed when growing tumor cells encounter a hypoxic microenvironment. The coupled effect can be less prominent when cancer cells prefer OXPHOS for ATP generation in nutrient-rich culture media (see Figure 4A,B). However, the dual functionality (positive and negative coregulation) can clearly be distinguished when KMT2A shRNA knockdown cells are subjected to metabolic stress tests. This transcriptional plasticity is further shown in CTCs that exhibit both high OXPHOS and glycolysis activities (Figure 6C—left panel), compared with primary tumors that dominantly utilize the latter (Figure 6C—right panel). In addition, we demonstrated that CTCs exploit the reserve functions of both pathways for survival in an in vitro circulation experiment.

A close-proximity interaction between a MAP and menin is essential for metabolic adjustment. Specifically, KMT2A catalyzes H3K4 trimethylation for open promoter chromatin to allow adjacent menin to recruit RNA polymerase II and other transcription factors to bind to the region [42,43]. Therefore, MI-503-mediated disruption of menin-KMT2A leads to an abolishment of OXPHOS gene transcription. As a result, decreased processing and synthesis of related proteins may lead to defects in OXPHOS complexes and their function for ATP generation. The tethering of menin-KMT2A is also important for transcription regulation of glycolytic genes. In the presence of MI-503, we also observed a decrease in glycolytic transcription activities, which likely lead to a reduced level of glycolytic enzymes. However, this transient inhibition is less impactful for glycolysis than OXPHOS. One possible explanation points to the required structural assembly of multiple OXPHOS complexes as a functional unit in the electron transport chain [44] while glycolytic enzymes operate independently to catalyze their corresponding intermediates for energy production. Therefore, although the MI-503 treatment greatly reduces ATP production from OXPHOS, existing glycolytic enzymes continue to operate and are capable of compensating for the energy loss.

## 4. Materials and Methods

### 4.1. Cell Cultures, shRNA Knockdown, and MI-503 Treatment

Human breast cancer cell lines T47D and MCF-7 were obtained from the ATCC and cell authentication was conducted at the ATCC using short tandem repeat DNA profiling. MISSION shRNA plasmids, sh*MEN1* (TRCN0000040141), sh*KMT2A* (TRCN0000005956), sh*MED12* (TRCN0000018578), sh*WAPL* (TRCN0000167548), and sh*GATA3* (TRCN0000019301), were obtained from Sigma-Aldrich (sequences listed in Table S2). Vehicle plasmid pLKO.1-puro (Addgene plasmid #8453) was obtained from Addgene. Plasmids were purified by QIAprep Spin Miniprep kit (Qiagen), and 293T cells were cotransfected with 3^rd^ Generation Packaging Mix (Applied Biological Materials, Richmond, BC, Canada) combined with either the shRNA plasmid or the vehicle plasmid for lentivirus production. The lentivirus-containing medium was harvested after 48 h of transfection, and cells were infected with lentivirus for 24 h. The infected cells were selected using puromycin at 4 μg/mL for 1 week. MI-503 (Selleckchem, Huston, TX, USA) was dissolved in dimethyl sulfoxide (DMSO) as a 10 mM stock and diluted to the working concentrations in the medium.

### 4.2. RNA-seq and Quantitative Reverse Transcription PCR (RT-qPCR)

Total RNA was isolated by PureLink RNA Mini Kit (Thermo Fisher Scientific, Waltham, MA, USA) with two biological replicates. Sequencing of cDNAs was performed with Illumina HiSeq3000, and the paired-end 100 bp FASTQ files were generated. We used STAR alignment software to align the sequencing data with the human reference genome GRCh38, and RSEM software to quantify the expression levels as fragments per kilobases of transcript per million (FPKM) mapped reads. Upregulated and downregulated genes were defined by a fold change ≥1.5 and ≤0.66, respectively, in sh*MEN1* knockdown cells relative to vehicle controls after filtering out the low expressed genes (FPKM values < 10). The pathway enrichment analysis was performed by the Database for Annotation, Visualization, and Integrated Discovery (DAVID, https://david.ncifcrf.gov). The heat maps were generated using log_2_ (fold change) based on normalized FPKM values. For RT-qPCR, the total RNA was isolated by the PureLink RNA Mini Kit (Thermo Fisher Scientific, Waltham, MA, USA) and the complementary DNA (cDNA) was made from 1 μg of total RNA by high-capacity cDNA reverse transcription kits (Applied Biosystems, Foster City, CA, USA). The primers of qPCR were listed in Table S3 (F1 and R1). qPCR quantification was performed by LightCycler 480 SYBR Green I Master (Roche, Basel, Switzerland) with 3 biological repeats, and relative expression levels of genes were calculated using the 2^−ΔΔCt^ method.

### 4.3. Proximity-Dependent Biotin Identification (BioID) Assay

Cloning and construction of the Tet-on inducible BioID construct were previously described [21]. Briefly, the pRetroX-mycBioID-MCS vector was cloned from the pcDNA3.1mycBioID plasmid (Addgene plasmid #35700) and pRetroX-Tight-Pur (Clontech). The full-length human *MEN1* cDNA from pBABE hygro MEN1 WT (Addgene plasmid #11024) was cloned into pRetroX-mycBioID-MCS at Not1 and Mlu I site by the Gibson reaction (New England Biolabs, Ipswich, MA, USA) to obtain pRetroX-mycBirA-MEN1. Engineered parental T47D and MCF7 cell lines that stably express a Tet repressor were infected with mycBirA-MEN1 retrovirus and stable cell lines were obtained under the selection of G418 (600 μg/mL for T47D and 300 μg/mL for MCF-7) and puromycin (1 μg/mL). To induce mycBirA-menin protein expression, stable T47D and MCF-7 cells were incubated in the medium with 2 μg/mL of doxycycline for 24 h and then in the medium with 50 mM biotin for another 24 h. For WES, the total lysate was collected by the RIPA buffer (Thermo Fisher Scientific, Waltham, MA, USA) containing the protease/phosphatase inhibitor (Thermo Fisher Scientific, Waltham, MA, USA); the cytoplasmic and nuclear lysates were isolated by NE-PER™ Nuclear and Cytoplasmic Extraction Reagents (Thermo Fisher Scientific, Waltham, MA, USA). For LC–MS/MS, cells were lysed by the RIPA buffer containing protease/phosphatase inhibitors. Free biotins were filtered out by 10 kDa filters (Sigma-Aldrich, St. Louis, MO, USA) and the biotin-labeled proteins were captured by incubation with 200 μL of streptavidin magnetic beads (Thermo Fisher Scientific, Waltham, MA, USA) for 5 h at 4 °C and collected by a magnetic stand. The beads were subject to 5 washes with 1 mL of RIPA buffer for 10 min at 4 °C and then 4 washes with PBS for 10 min at room temperature (RT). About 10% of the PBS-beads mixture was boiled for 10 min to release the proteins and the supernatant proteins were analyzed using WES. After removing the supernatant from the remaining mixture, beads were kept at −80 °C until further mass spectrometry analysis in the Proteomics Core at the Sanford-Burnham Prebys Medical Discovery Institute.

The total sample peptide amount was evaluated by Pierce Quantitative Colorimetric Peptide Assay, 500 Assays (Thermo Fisher Scientific, Waltham, MA, USA) before loaded into LC–MS. To reconstitute dried samples, 2% acetonitrile and 0.1% formic acid were used and reconstituted samples were analyzed by LC–MS/MS using a Proxeon EASY nanoLC system (Thermo Fisher Scientific, Waltham, MA, USA) coupled to an Orbitrap Elite mass spectrometer (Thermo Fisher Scientific, Waltham, MA, USA). An analytical C18 Acclaim PepMap column (75 µm × 500 mm, 2 µm particles; Thermo Fisher Scientific) was used to separate peptides at a rate of 300 nL min^−1^ for over 121 min: 1–6% B in 1 min, 6–23% B in 56 min, 23–34% B in 37 min, 34–48% B in 26 min, and 48–98% B in 1 min (A = FA 0.1%; B = 80% ACN: 0.1% FA).

Operation of the mass spectrometer was set up in data-dependent acquisition mode. Spectra of the first spectrometer (MS1) were measured by Orbitrap analyzer with a resolution of 60,000 (automatic gain control (AGC) target of 1e6 and a mass range from 350 to 1450 m/z). Collision-induced dissociation method was used to generate fragments for the second spectrometer (MS2) and data was acquired by the linear ion trap (AGC target of 1e4, isolation window of 2 m/z, and a normalized collision energy of 35). The duration of dynamic exclusion was enabled with 30 s.

MaxQuant software (v1.5.5.1) was used to process MS raw files with most of the default settings, and the Andromeda search engine was used to search against the human UniProt database and GMP cRAP sequences (the common contaminants database). Variable modifications were set to methionine oxidation and N-terminal acetylation, while fixed modification was set to carbamidomethylation. The minimum ratio count was set to two for label-free protein quantification, and peptides for quantification were set to unique and razor. Proteins and peptides were selected based on the false discovery rate (FDR) set to 0.01, with a minimum length of seven amino acids.

We analyzed the label free quantitation (LFQ) intensity and defined the biotinylated differential proteins (menin associated proteins, MAPs) as ≥3-fold in doxycycline^+^/biotin^+^ cells compared to the doxycycline^+^/biotin^−^ cells’ counterpart. The heat map was generated using log(LFQ intensity+1). The network analysis was performed by R package *igraph* and the distance was defined as the ratio between the LFQ intensity of each MAPs and menin.

### 4.4. WES and Immunoprecipitation Assays

Antibodies were obtained commercially and the dilutions were used as the following: menin (Bethyl Laboratories, A300-105A; 1:100), KMT2A (Bethyl Laboratories, A300-374A; 1:10), GATA3 (Cell Signaling Technology, 5852S; 1:25), WAPL (Cell Signaling Technology, 77428S; 1:50), MED12 (Cell Signaling Technology, 4529S; 1:25), LaminA/C (Cell Signaling Technology, 2023S; 1:50), GAPDH (Cell Signaling Technology, 2118S; 1:200), and β-Actin (R&D System, MAB8929; 1:100). For WES, 0.5 μg/μL of protein lysate was used for one reaction. Menin, KMT2A, MED12, WAPL, GATA3, LaminA/C, and GAPDH were detected by the Anti-Rabbit HRP Detection module (ProteinSimple, San Jose, CA, USA) or by 1× Anti-Rabbit HRP conjugate (ProteinSimple, San Jose, CA, USA) in the Anti-Mouse HRP Detection module (ProteinSimple, San Jose, CA, USA) when combined with β-Actin. β-Actin was detected by the Anti-Mouse HRP Detection module (ProteinSimple, San Jose, CA, USA). For detection of biotin labeled proteins, total lysates were visualized by the streptavidin-HRP and chemiluminescent substrate (ProteinSimple, San Jose, CA, USA). The WES assays were run with Jess/Wes 12–230 kDa Pre-filled Plates or Jess/Wes 66–440 kDa Pre-filled Plates (ProteinSimple, San Jose, CA, USA) dependent on the molecular weights and analyzed by Compass for SW software (ProteinSimple, San Jose, CA, USA). The original WES images can be found at Figure S8.

For immunoprecipitation, T47D and MCF-7 cells were treated with DMSO or 1 μM of MI-503 for 3 days. The nuclear lysates were incubated with the menin antibody (Bethyl Laboratories, A300-115A) or rabbit IgG (kch 504250, Diagenode) at 4 °C for 18 h, and the antibody was captured by Protein A/G magnetic beads (Thermo Fisher Scientific, Waltham, MA, USA) at RT for 2 h. Then, the beads were washed with 1 mL of TBST (25 mM Tris, 0.15 M NaCl, and 0.05% Tween-20, pH 7.5), twice with 1 mL of NETN buffer (50 mM Tris-HCl pH 8, 150 mM NaCl, 1% NP-40, and 1 mM EDTA), and finally with 1 mL of ddH_2_O. For elution, the beads were resuspended with 30 μL of RIPA buffer containing 2% SDS and protease/phosphatase inhibitors at RT for 10 min, and the supernatant was separated from beads by a magnetic stand.

### 4.5. Seahorse Assays

Cells were seeded on the cell culture microplate (Agilent, Santa Clara, CA, USA) with 12,000 cells per well and cultured to reach 90% confluent. For the Seahorse mitochondrial stress test and real-time ATP rate test, cells were preincubated in pH 7.4 Seahorse DMEM media (Agilent, Santa Clara, CA, USA) containing 10 mM glucose, 2 mM glutamine, and 1 mM pyruvate in a non-CO_2_ incubator at 37 °C for 1 h. For the Seahorse glycolysis stress test, cells were preincubated in pH 7.4 Seahorse DMEM media containing 1 mM glutamine in an incubator with the same settings for 1 h. For Seahorse assays after circulation, cells were resuspended in either Seahorse mitochondrial stress or Seahorse glycolysis stress media. Thereafter 1500 cells were seeded per well on the cell culture microplate (Agilent, Santa Clara, CA, USA) and was set at RT for 20 min followed by incubation at 37 °C in a non-CO_2_ incubator for 20 min. The sensor cartridge (Agilent, Santa Clara, CA, USA) was rehydrated 1 day before the assay and the inhibitors were serially added just before the assay. The final concentrations of chemicals used were as the following: for the mitochondrial stress test, pore A: 1 μM oligomycin; pore B: 1 μM FCCP; and pore C: rotenone and antimycin A, 0.5 μM each. For the glycolysis stress test, pore A: 10 mM glucose; pore B: 1 μM oligomycin; and pore C: 5 mM 2-DG. For the real-time ATP rate test, pore A: 1.5 μM oligomycin and pore B: rotenone and antimycin A, 0.5 μM each. The assays were performed by a Seahorse XFe96 Analyzer (Agilent, Santa Clara, CA, USA) and analyzed by Seahorse XFe96 Analyzer Wave 2.4 (Agilent) software. For normalization, Hoechst 33,342 (Thermo Fisher Scientific, Waltham, MA, USA) was combined to add in the last injection at the final concentration of 20 μM to stain the cell nucleus, and the images were taken by Cytation1 (BioTek, Winooski, VT, USA) with Agilent Seahorse XF Imaging and Cell Counting software. For validating the ATP production measurement by Seahorse XFe96 analyzer, a comparative quantitation was performed by the CellTiter-Glo Luminescent Cell Viability Assay Kit (Figure S7).

### 4.6. Circulating Tumor Cells (CTCs) Isolation and Culture

Blood samples were collected from advanced breast cancer patients according to the IRB protocol approved by the University of Texas Health Science Center at San Antonio (CTRC# 07-32). About 15 mL of Ficoll (GE Healthcare Life Sciences) was placed in a 50 mL conical tube, and 8 mL of the blood sample was carefully added on the top of the Ficoll. CTCs remained in the interphase peripheral blood mononuclear cells (PBMCs) layer after centrifugation at 400× *g* at RT for 30 min. The PBMC layer was carefully moved into a 15 mL conical tube and mixed with 10 mL of the MojoSort buffer (Biolegend, San Diego, CA, USA). After centrifugation again at 200× *g* for 5 min at 4 °C, CTCs were cleaned by the MojoSort buffer. Then, CTCs were cultured and expanded in the PRIME-XV tumorsphere medium (Fujifilm Irvine Scientific, Santa Clara, CA, USA) for 1 week for enrichment. For collection, cells were dispersed and washed by the MojoSort buffer and contaminated blood cell depletion was carried out using 10 μL of Human CD45-nanobeads (Biolegend, San Diego, CA, USA). CD45^−^ CTCs were harvested in the supernatant by a magnetic stand after a few washes and resuspensions with the MojoSort buffer.

### 4.7. Immunofluorescence Analysis

CTCs were fixed with 4% paraformaldehyde (Santa Cruz Biotechnology) for 10 min and permeabilized with PBS containing 0.5% Triton X-100 for 5 min at RT. Nonspecific binding was blocked with 5% goat serum in PBS containing 0.1% Triton X-100 for 30 min at RT. Immunostaining was performed at 4 °C overnight with primary antibodies, followed by fluorescence-conjugated secondary antibodies at a concentration of 5 μg/mL (Thermo Fisher Scientific). Images were captured with the Zeiss LSM710 confocal microscope equipped with 64× NA 1.4 oil DIC (Carl Zeiss). Antibodies used for staining were as follows: rabbit anti-ALDH1 (bs-10162R, Bioss, 1:200), mouse anti-pan-CK (4545, Cell Signaling Technology, 1:3000), rabbit anti-EpCAM (ab71916, Abcam, 1:200), rabbit anti-NANOG (ab21624, Abcam, 1:800), mouse anti-OCT4 (ab184665, Abcam, 1:500), rat anti-CD44 (ab40983, Abcam, 1:400), mouse anti-CD45 (ab33533, Abcam, 1:200), goat anti-rabbit (A-11037), goat anti-rabbit (A-110340), and goat anti-mouse (A-11032).

### 4.8. Single-Cell qRT-PCR

Single CD45^−^/Calcein AM^+^ CTCs were retrieved manually and lysed in 4 μL of CellsDriect 2× reaction mix (Invitrogen) and the target genes were preamplified as described previously with modifications (26). The preamplification reaction contained 4 μL of single cell lysate, 1.8 μL of DNA suspension buffer (Teknova), 0.2 μL of SuperScript III RT/Platinum Taq mix (Invitrogen), and 1 μL of the premixed outer primer sets (F1 and R1, sequences were listed in Table S3, 500 nM per primer). The thermal cycle was 50 °C, 15 min; 95 °C, 2 min; 95 °C, 15 s; and 60 °C, 4 min, and amplicons were amplified for 20 cycles. Non-amplified DNA was removed by Exonuclease I (New England BioLabs) at 37 °C for 30 min. Real-time PCR reactions were run using BioMark^TM^ HD System (Fluidigm) with the semi-nested primer pair (Table S3, F1 and R2) and analyzed by Real-Time PCR Analysis Software (Fluidigm). The relative expression was normalized to the *ACTB* level to obtain ΔCt, and the log(−2^ΔCt^) was used as the relative gene expression for the t-SNE analysis and violin plots with R package *ggplot2*.

### 4.9. In Vitro Flow-Based Assay

Cell circulation was performed using an ibidi pump system (ibidi) with μ-slide I Luer 0.6 mm (ibidi). About one million cells in 10 mL of the medium were loaded in a perfusion set held on a fluidic unit. The fluidic unit was incubated in a humidified incubator aired with CO_2_ at 37 °C, and the flow rate was 16.67 mL/min with 5 s switching time controlled by PumpControl Software. After circulation, cells were collected and centrifuged at 500× *g* for 5 min. The cell pellet was suspended and washed in PBS before cell viability assays and Seahorse assays.

### 4.10. Cell Viability Assay

Cell viability assay was performed by the CellTiter-Glo Luminescent Cell Viability Assay Kit (Promega). For MI-503 treatment, T47D or MCF-7 cells were seeded in 96-well plate with 200 cells per well and treated with DMSO or MI-503 for 3 days. For circulated cells, 2000 cells after circulation were seeded in 50 μL of medium in 96-well plates. The assay reagent mixture was directly added into the cell medium at a 1:1 ratio for 15 min to lyse the cells, and the luminescent signal was detected using the Thermo Luminoskan Ascent Microplate Reader (Thermo Fisher Scientific).

### 4.11. In Silico Analysis

RNA-seq data of primary tumors and normal controls in The Cancer Genome Atlas (TCGA) breast cancer cohort were downloaded from Broad Genome Data Analysis Center (GDAC) Firehose. Expression levels of *MEN1*, MAPs, and glycolytic and OXPHOS genes were extracted, and Pearson correlation coefficients of the expression of individual *MEN1/*MAP genes and individual OXPHOS/glycolytic genes were calculated and plotted as heatmaps with R package *heatmap.2.* Mean expression levels of OXPHOS and glycolytic genes were plotted against the expression of *MEN1* and the 4 MAP genes (*KMT2A*, *MED12*, *WAPL*, and *GATA3*). Violin plots, dot plots, and scatter plots were produced with R package *ggplot2*. A Kaplan–Meier survival analysis was performed using R package *survminer* on ERα-positive and ERα-negative patients, separately, of the METABRIC dataset downloaded from the cBioPortal. High expression levels of *MEN1* were defined as top 20%, and low expression levels bottom 20%. Segmented copy number values > 0 was considered as amplification, while <0 as deletion. ChIP-seq data was obtained from Gene Expression Omnibus database (GEO number GSE85317) [10] and used EaSeq to analyze and generate peaks.

### 4.12. Data Availability

The RNA-seq data in this study is available through the Gene Expression Omnibus (GEO) under accession number GSE145955.

## 5. Conclusions

In conclusion, we demonstrated that menin has a previously uncharacterized role in metabolic energy arbitration, in addition to modulating DNA replication and repair and cellular transformation [45,46]. The mode of menin-regulated transcription can further be adjusted by a group of MAPs. We propose that MAPs are transcriptional sensors to determine a preference between glycolysis and OXPHOS. Upstream metabolic signaling modulators (e.g., MYC, PGC-1α, AMPK, HIF-1α, mTOR, and RAS) [8,30,32,47,48] may synergize with menin and MAPs for transcription modulation. To fuel energy for proliferation and differentiation, this coordinate regulation is essential for cells to assess available sources (i.e., oxygen, glucose, or free amino acid) for ATP generation.

## Supplementary Materials

The following are available online at https://www.mdpi.com/2072-6694/12/9/2715/s1, Figure S1: Increased *MEN1* expression and copy number are related to poor prognosis of ERa+ breast cancer patients; Figure S2: The BioID system, network analysis of 35 MAPs in T47D and in silico analysis of anti-menin and anti-KMT2A ChIP-seq; Figure S3: Expression correlation relationship of MAPs and OXPHOS/glycolytic genes; Figure S4: Gene and protein expressions and extracellular-flux analysis in MAP shRNA knockdown cells; Figure S5: Gene expressions and extracellular-flux analysis in cells treated with MI-503; Figure S6: CTCs isolation and in vitro circulation system; Figure S7: Validation of ATP quantitation by Seahorse Seahorse XFe96 analyzer using CellTiter-Glo Luminescent Cell Viability Assay Kit; Figure S8: The original WES images; Table S1: Biotin labelled nuclear proteins in T47D and MCF-7 cells (also see Figure 2C); Table S2: shRNA sequences used in this study; Table S3: RT-qPCR and Bio-mark primers used in this study.
