# Supplementary material for: Menin and Menin-Associated Proteins Coregulate Cancer Energy Metabolism
Source: Cancers (Basel). 2020 Sep 22;12(9):2715. doi: 10.3390/cancers12092715 (PMC7564175; doi:10.3390/cancers12092715)
Supplement: Supplementary file 1 [file cancers-12-02715-s001.pdf]

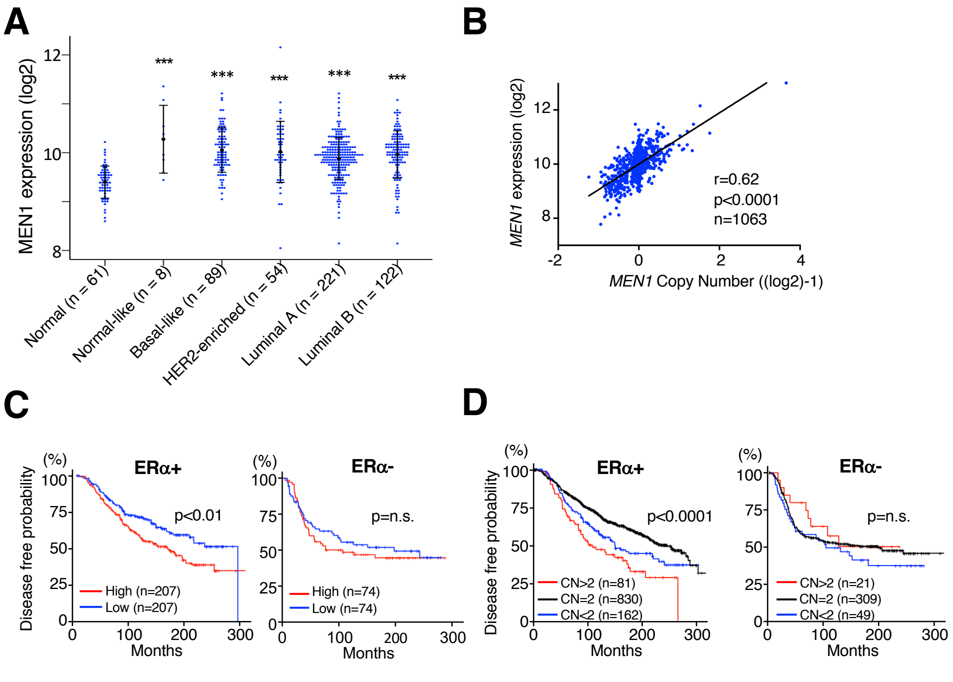

Figure S1.

Increased *MEN1* expression and copy number are related to poor prognosis of ERα+ breast cancer patients. (**A**) *MEN1* expression in different breast cancer PAM50 subtypes in TCGA breast cancer cohort. Significant levels were based on Duncan's multiple range tests (****P* < 0.001). (**B**) Scatter plot and linear regression of *MEN1* expression and copy number in TCGA breast cancer cohort. Significant levels were based on Pearson correlation coefficient. (**C**) and (**D**) Kaplan-Meier survival analyses and significant levels were based on logrank tests from Molecular Taxonomy of Breast Cancer International Consortium (METABRIC) cohort based on *MEN1* expression (**c**) or copy number (**D**).

A

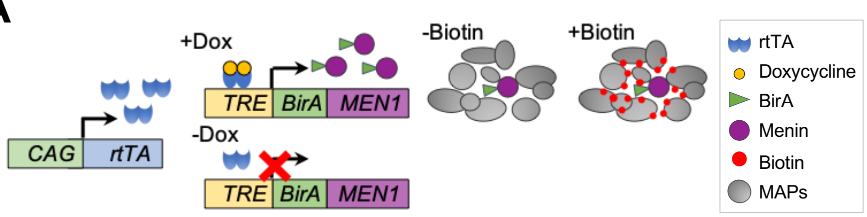
+Dox
TRE
BirA
MEN1
-Dox
TRE
BirA
MEN1
CAG
rtTA
-Biotin
+Biotin
rtTA
Doxycycline
BirA
Menin
Biotin
MAPs

B

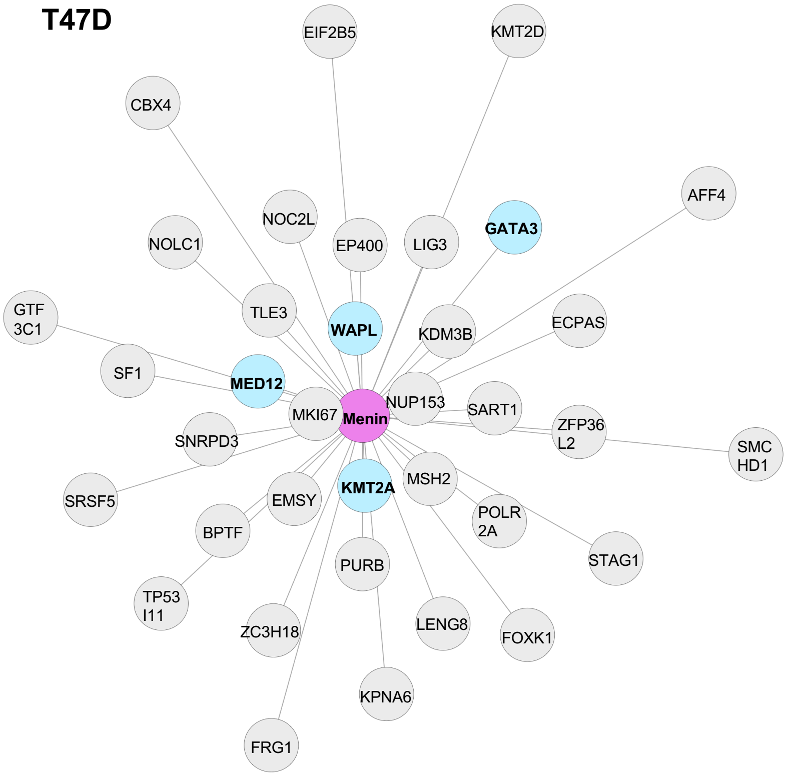
T47D
EIF2B5
KMT2D
CBX4
AFF4
NOC2L
NOLC1
EP400
LIG3
GATA3
GTF 3C1
TLE3
WAPL
KDM3B
ECPAS
SF1
MED12
NUP153
SART1
MKI67
Menin
ZFP36 L2
SMC HD1
SNRPD3
MSH2
KMT2A
EMSY
POLR 2A
SRSF5
BPTF
STAG1
PURB
TP53 I11
ZC3H18
LENG8
FOXK1
KPNA6
FRG1

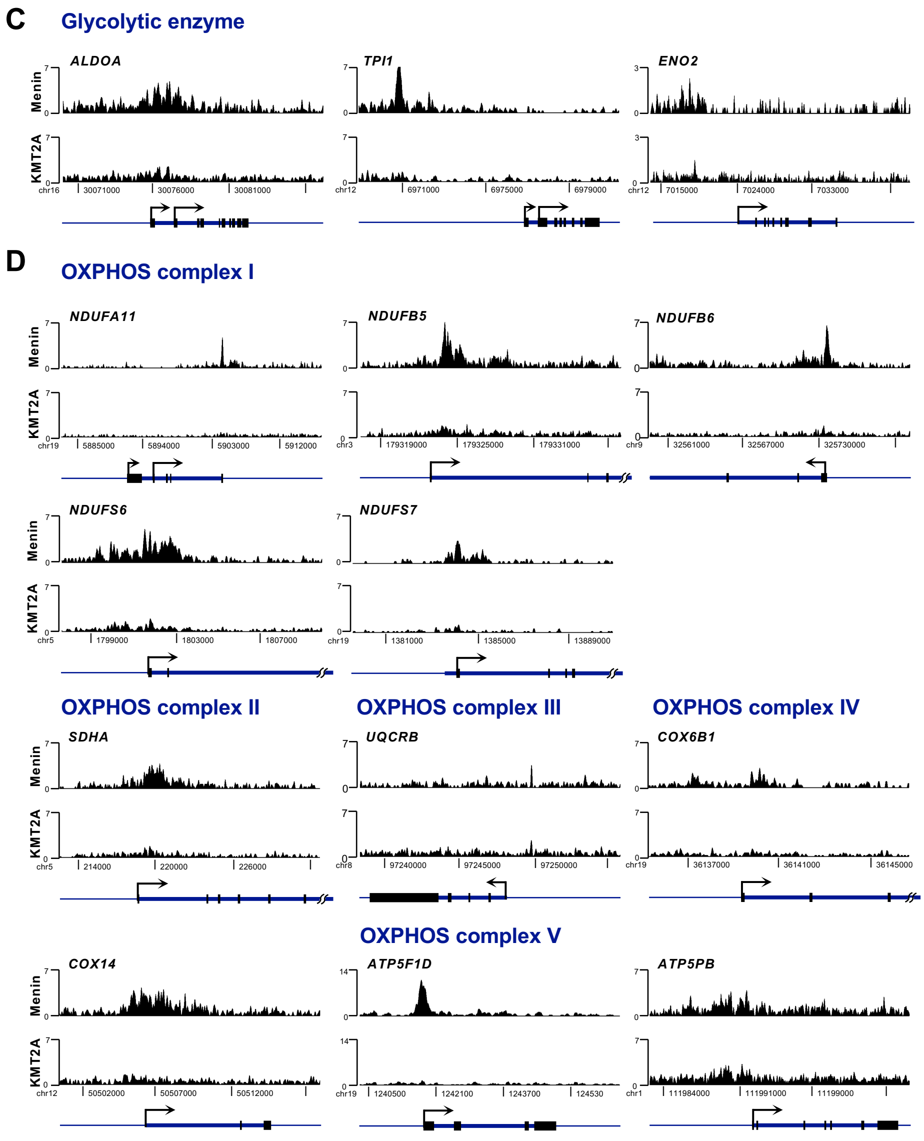

Figure S2.

The BioID system, network analysis of 35 MAPs in T47D and *in silico* analysis of anti-menin and anti-KMT2A ChIP-seq. (**A**) Schematic illustration of biotin labeling of MAPs using BioID proximity-dependent biotinylation with a doxycycline-inducible biotin ligase *BirA-MEN1* fused gene. (**B**) Network analysis of 35 MAPs in T47D cells. The distance between menin and MAPs represented the protein quantitative ratio of each MAP and menin. MAPs marked in blue were further validated by WES. (**C**) *In silico* analysis of anti-menin and anti-KMT2A ChIP-seq data indicates that anti-Menin immunoprecipitation enriched the promoter and gene body DNA sequence of the representative glycolytic genes and (**D**) OXPHOS genes in MCF-7 cells.

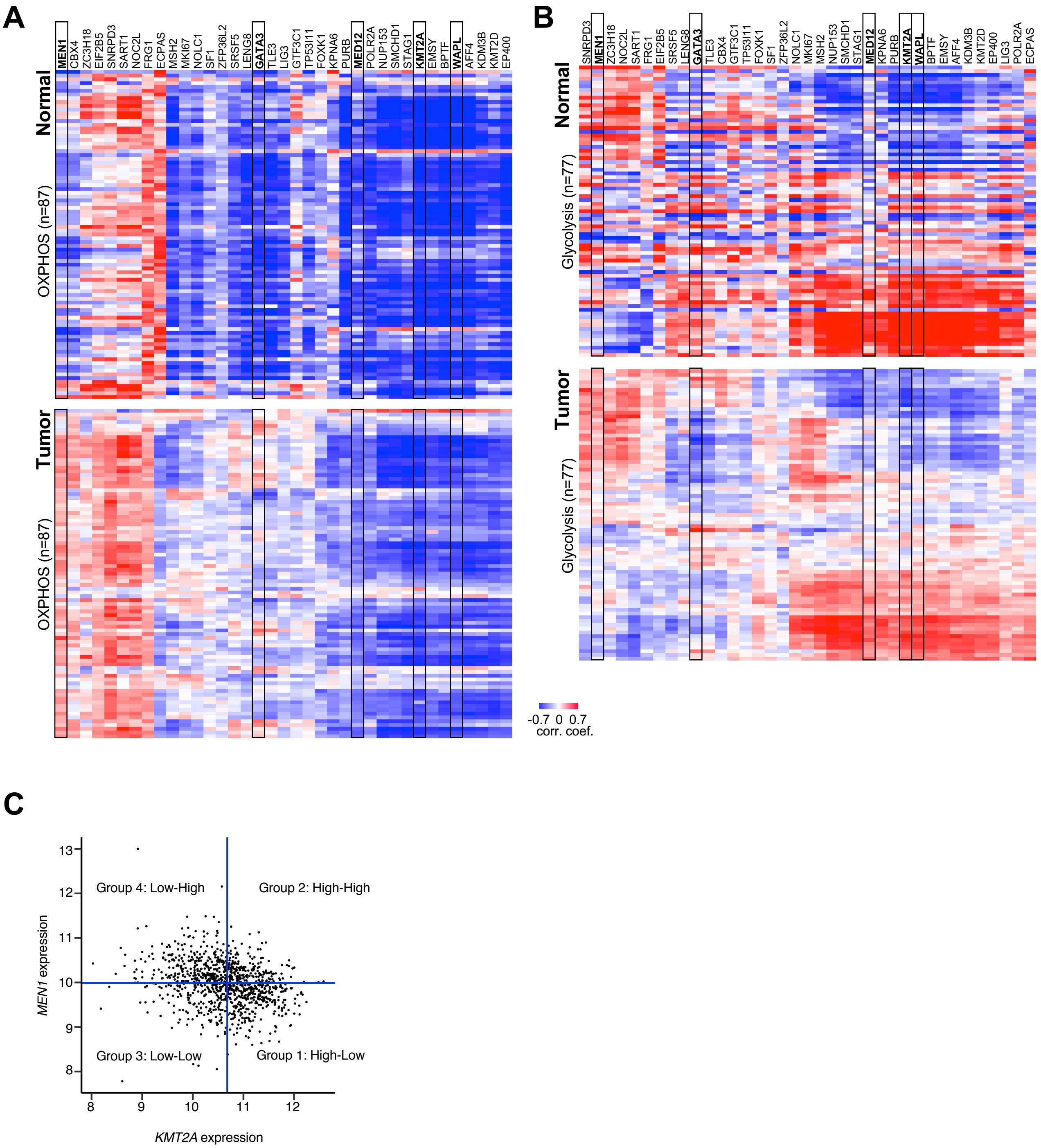

Figure S3.

Expression correlation relationship of MAPs and OXPHOS/glycolytic genes. (**A**) and (**B**), Heatmaps of gene expression correlations of 37 MAPs (including *MEN1*) genes with OXPHOS genes (**A**) or glycolytic genes (**B**) in normal and tumor samples from TCGA breast cancer cohort. (**C**) Scatter plot of MEN1 and KMT2A expression in TCGA breast tumors. The tumors are grouped into 4 groups (1-4) as in Figure 3E according to median values of both genes' expression. Similar groupings are also applied based on the median expression of *MEN1* and the other three MAP genes (*MED12*, *WAPL* and *GATA3*).

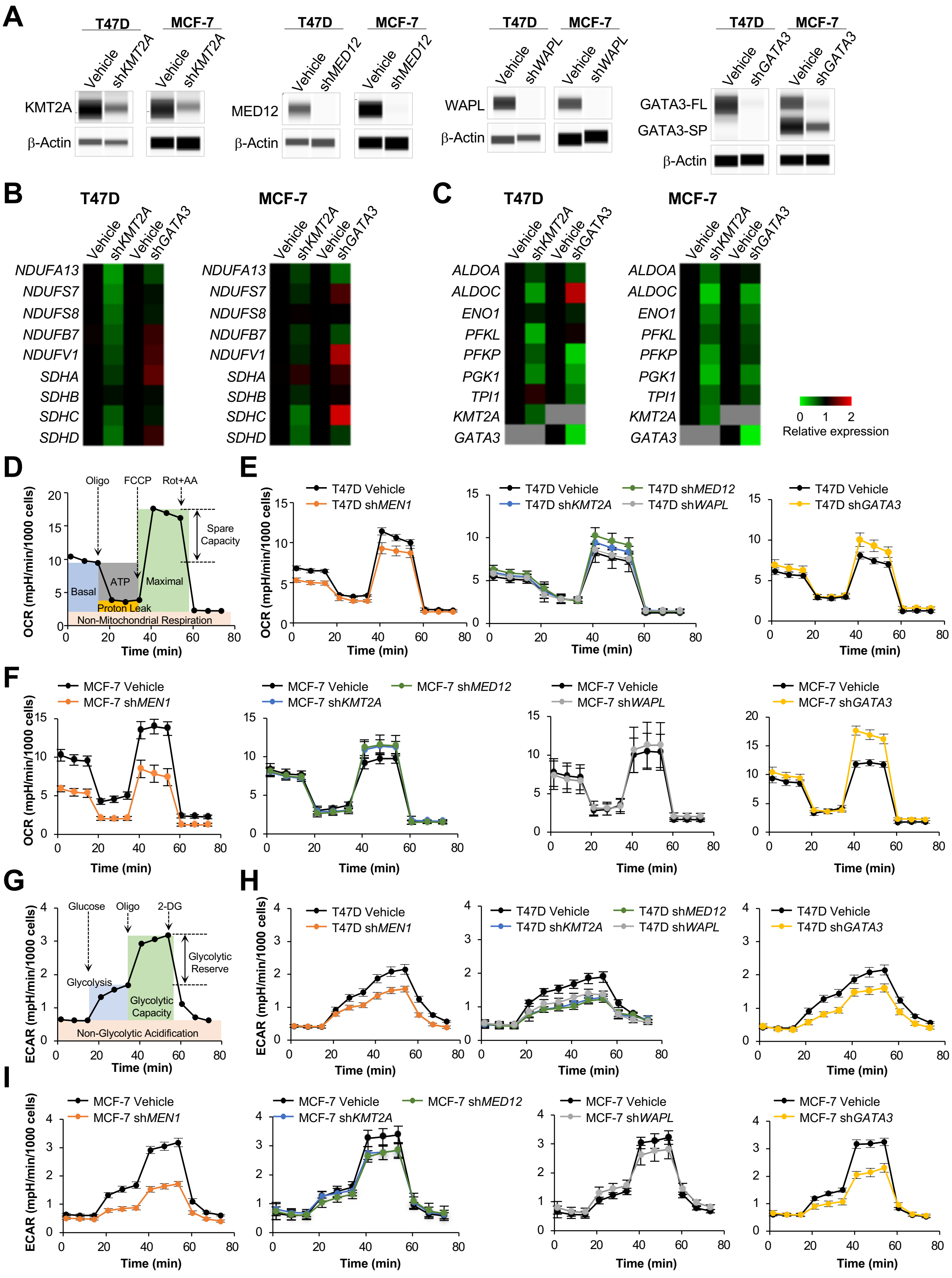

Figure S4.

Gene and protein expressions and extracellular-flux analysis in MAP shRNA knockdown cells. (**A**) WES of total lysate from T47D or MCF-7 cells subject to vehicle or MAP shRNA lentivirus infection. (**B**) and (**C**) Heat maps of RT-qPCR of OXPHOS genes (**B**) or glycolytic genes (**C**) in T47D or MCF-7 cells. The expression in vehicle control is normalized as 1 (n = 3). (**D**) Seahorse mitochondrial stress test profile. (**E**) and (**F**) Line charts of OCR for mitochondrial functions in T47D (**E**) and MCF-7 (**F**) cells. (**G**) Seahorse glycolytic stress test profile. (**H**) and (**I**) Line charts of ECAR for glycolytic functions in T47D (**H**) and MCF-7 (**I**) cells. Data are presented as mean±S.D. (n = 15-20 technical-replicate wells).

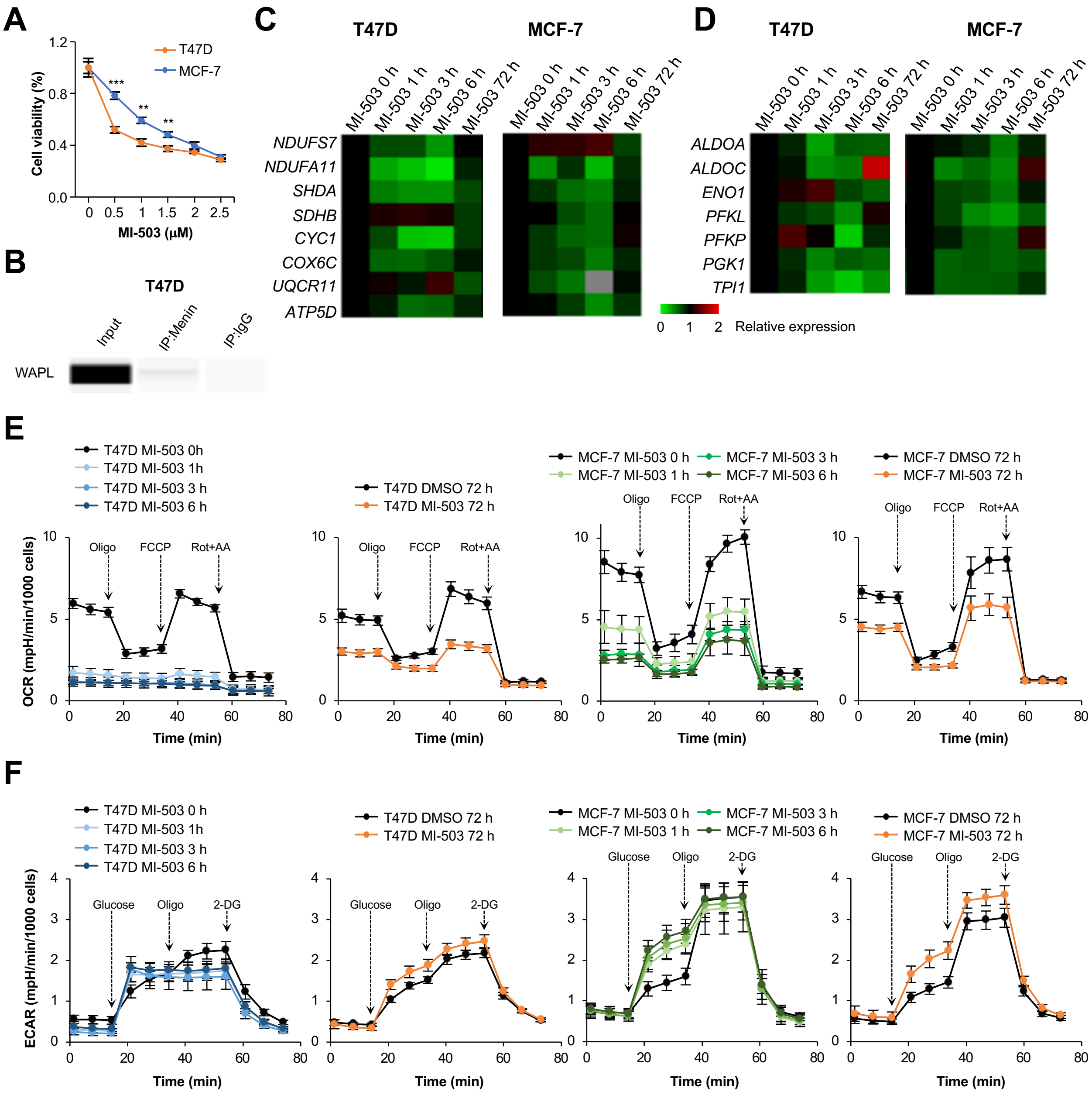

Figure S5.

Gene expressions and extracellular-flux analysis in cells treated with MI-503. (**A**) Cell viability of T47D and MCF-7 cells under different dosages of MI-503 for 3 days (n= 5). (**B**) Co-immunoprecipitation of WAPL, another MAP, with menin pulled down by anti-menin but not anti-IgG. (**C**) and (**D**) Heat maps of relative expression of OXPHOS (**C**) and glycolytic (**D**) genes by RT-qPCR in T47D or MCF-7 cells subject to 1 μM MI-503 for 0, 1, 3, 6, 72 h, or DMSO for 72 h. Gene expressions post MI-503 treatment for 1, 3, and 6 h were normalized to that of the 0 h control; gene expressions post MI-503 treatment for 72 h were normalized to that of DMSO treatment for 72 h. The expressions in 0 h of MI-503 treated cells and 72 h of DMSO treated cells were normalized as 1 (n = 3). (**E**) and (**F**) Line charts of extracellular-flux analysis for mitochondrial (**E**) and glycolytic (**F**) functions in T47D or MCF-7 cells treated by MI-503 for 0, 1, 3, 6, 72 h, or DMSO for 72 h. Data are presented as mean ± S.D. (n = 15-20 technical-replicate wells).

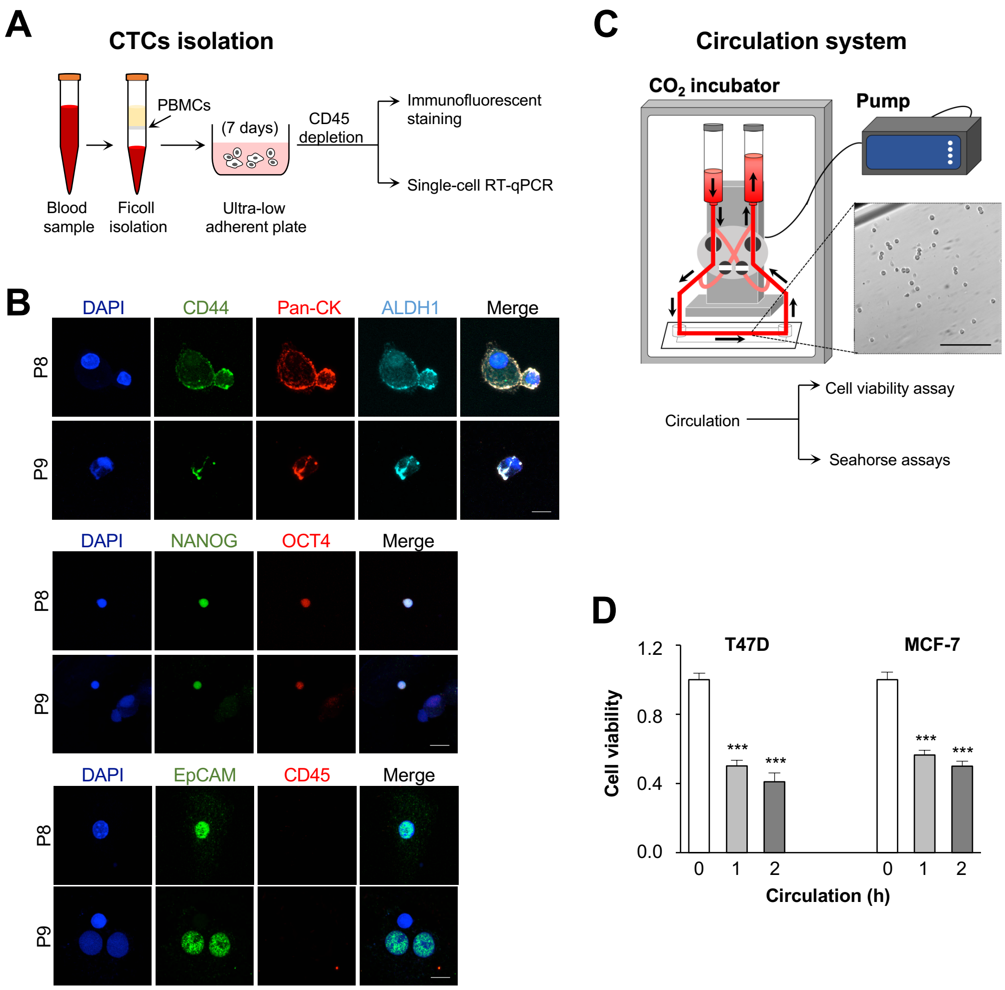

Figure S6.

CTCs isolation and *in vitro* circulation system. (**A**) Schematic workflow of CTC isolation, expansion, and subsequent experiments. (**B**) Representative images of CTCs staining with stem cell or epithelial markers. Scale bar, 10 μm. (**C**) Scheme of the circulation system and experimental assays. (**D**) Bar chart of cell viability of T47D and MCF-7 cells after circulation (n = 5 technical-replicate wells). Data are presented as mean ± S.D. Unpaired two-tailed Student's *t*-test was used for statistical significance determination of 0 h and each treatment (****P* < 0.001).

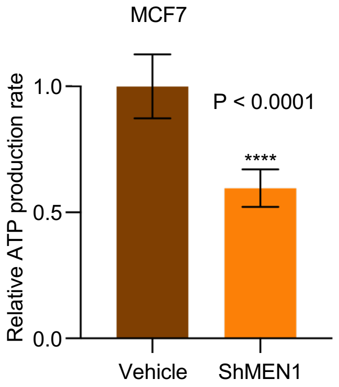

Figure S7.
Validation of ATP quantitation by Seahorse Seahorse XFe96 analyzer using CellTiter-Glo Luminescent Cell Viability Assay Kit. ATP reduction is observed in MCF7 cells treated with menin shRNA, consistent with the measurement result by Seahorse in Fig. 4A.

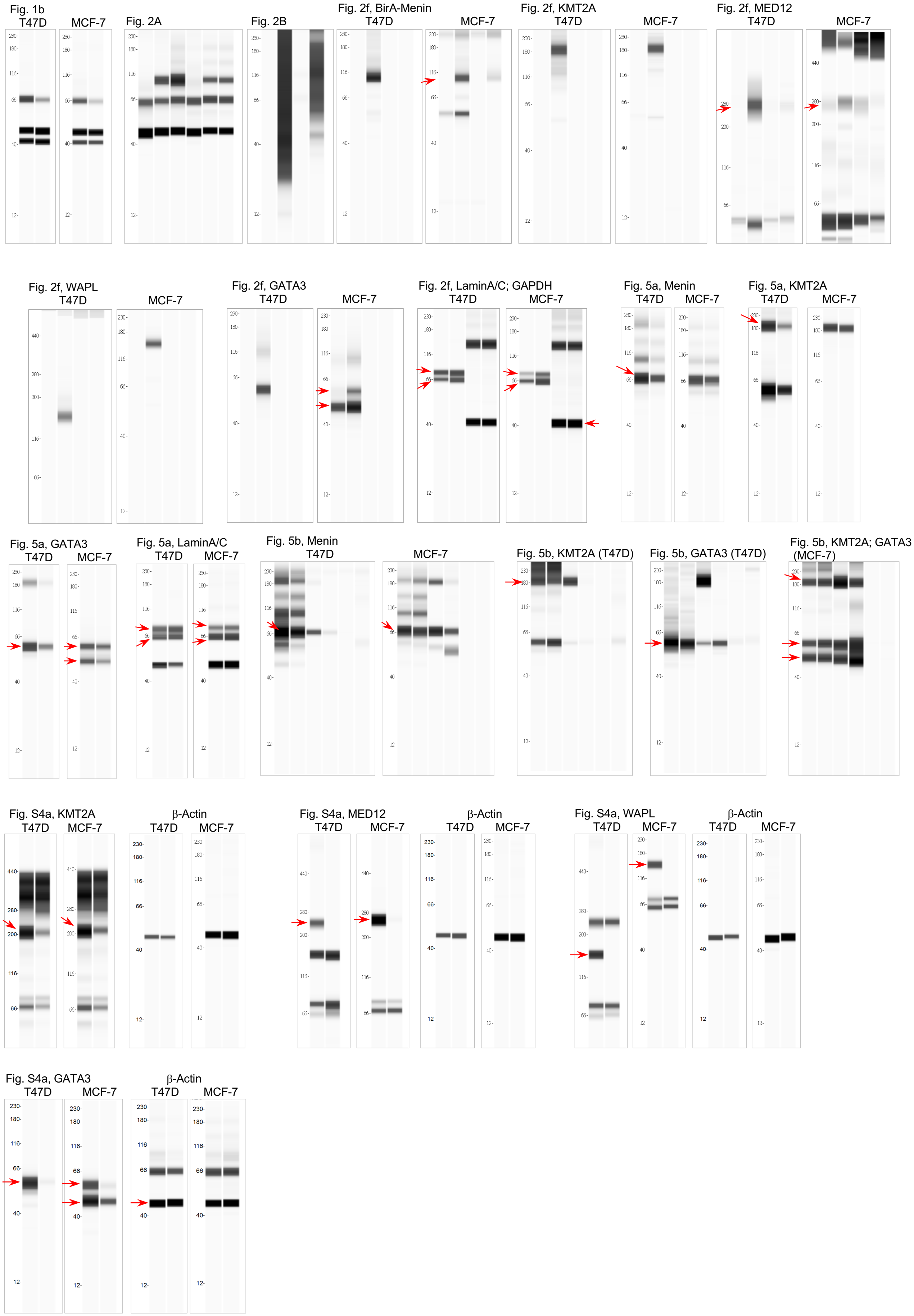

Figure S8. The original WES images.

Table S1. Biotin labelled nuclear proteins in T47D and MCF-7 cells. Related to Figure 2C.

	**T47D**	**MCF-7**
**Class 1: Basal transcription factor complexes**	ACIN1, AFF4, BPTF, BTAF1, CCNT1, CHD8, DHRS7B, EIF2B5, EIF3C, ELMSAN1, GTF2F2, GTF3C1, HP1BP3, INTS1, INTS9, LIG3, MED1, MED12, MED14, NELFE, NOC3L, ORC2, PAF1, PAPOLA, POLA1, POLR2A, POLR2B, RFC5, SENP3, SUPT16H, TAF7, TOX4, WAC, WDR82, ZC3H4, ZHX3, ZMYND8.	AFF4, BPTF, CHD1, CHD3, EIF2B5, EIF5B, GRHL2, GTF3C1, HCFC1, HIRA, INTS8, LIG3, MED12, MED24, NCOR1, POLR1D, POLR2A, POLR2E, SMC6, WDHD1.
**Class 2: DNA repair complexes**	ARID1B, BAZ1B, DCAF13, DDB1, EMSY, MSH2, NBN, PMS2, RBBP8, RIF1, SSBP1, XRCC1.	ARID1A, EMSY, FANCI, MDC1, MSH2, TNKS1BP1.
**Class 3: mRNA modeling; RNA helicase; spliceosomes**	C17orf85, C19orf43, CMTR1, CPSF6, DDX10, DDX23, DDX27, DDX41,DDX42, DDX46, DDX54, DDX56, DHX36, DHX38, DHX40, DIS3, EIF2AK2, FIP1L1, FRG1, FUBP1, MCM3AP, MRTO4, NHP2L1, NUFIP2, PARN, PLRG1, PRCC, PRPF31, PRPF6, RAE1, RBM10, RBM17, RBM26, RBM28, RBM7, SART1, SART3, SF1, SF3A1, SF3B5, SF3B6, SMN1, SNRPD1, SNRPD3, SRBD1, SRSF5, TFIP11, TTC37, WDR33, XAB2, ZFP36L2, ZNF638.	ATXN2L, CNOT2, CPSF2, DCP1A, EDC4, ESRP1, FRG1, GEMIN4, GEMIN6, KHDRBS1, KHSRP, PCID2, RANGAP1, SART1, SF1, SF3A2, SF3B2, SKIV2L2, SNRPD3, SRRM2, SRSF3, SRSF5, THOC1, THRAP3, TRA2A, UPF2, ZCCHC8, ZFP36L2.
**Class 4: Nuclear scaffold complexes relate**	DRG1, DRG2, ECM29, KPNA6, MISP, MKI67, NDC1, NSFL1C, NUP107, NUP153, NUP188, NUP205, NUP85, PPP1R10, SMC1A, SMC4, SMCHD1, SUN2, SURF6, TUBB6, TUBGCP2, WDR3, WDR46.	ECM29, EHD4, KPNA6, MAD1L1, MKI67, MYH10, NOP14, NUP153, NUP210, NUP88, PLK1, POM121C, SAFB2, SEC13, SMCHD1, SYMPK, TPX2.
**Class 5: Site-specific transcription factors**	CHAMP1, NCAPD2, NCAPD3, PDS5A, PDS5B, POGZ, STAG1, TOP1, TOP2B, WAPL.	STAG1, WAPL.
**Class 6: Cohesin related complexes and mediators**	ADNP, AQR, ARFGEF1, BDH1, CBX4, CRK, DNAJA3, FOXK1, FOXK2, GATA3, GREB1, JAK1, MKL2, MLLT11, MYBBP1A, NCOA6, NFIC, NKRF, PARP14, PEG10, PURB, RFX1, SBNO1, SCML2, SCYL1, SENP1, SIN3A, STAT2, TCF20, TLE3, TP53I11, YAP1, YY1AP1, ZC3H18, ZNF148, ZNF281, ZNF687.	CBX4, CHCHD3, DIDO1, ERBB2IP, FOXK1, G3BP1, GATA3, GATAD2B, GSE1, ILF3, MBD3, MYEF2, PPM1D, PURB, SAMD1, SPEN, TCF25, TLE3, TP53BP1, TP53I11, TRPS1, TSC2, ZC3H18, ZMYM2, ZNF384, ZNF644.
**Class 7: Histone modifying enzymes**	EP400, EZH2, GRWD1, HDAC2, JMJD1C, KDM2A, KDM3B, KMT2A, KMT2D, NOC2L, PHF8, RSBN1, RSBN1L, SETD1A, SMARCAD1, TRRAP, WIZ.	BAZ2A, EP400, HIST1H2BB, HIST1H2BC, KAT6B, KDM3B, KMT2A, KMT2D, NOC2L, OGT, PAXIP1, SAP130, SMARCAL1, SMARCC2, SRCAP, STK19, SUDS3.
**Class 8: tRNA modification, ubiquitination, and others**	ACOT13, BCLAF1, CRNKL1, CUL4B, CUL5, DARS2, DERA, EARS2, EBP, FAM120C, FBXL6, FDPS, GNL2, GPATCH1, IMP4, IPO11, ITPR2, KARS, KIF13B, L1RE1, LENG8, NFS1, NOL6, NOLC1, NOP2, PCNP, PDCD6, PPP2R5D, PPT1, PRKACG, PRKCD, PSPC1, QIL1, RANBP3, RBBP6, RNF213, RPF2, RPP30, RPP38, RSL1D1, SRP14, SUGT1, TAOK2, TCOF1, TRMT1L, TRMT5, TRNT1, UBE2L6, UBE3A, UBE4B, UBR4, UBXN7, UGDH, UNC45A, UTP6, WDR75, YARS2, YIF1B, YLPM1.	AP3D1, BAG2, BOLA2, CBS, DUT, IMPDH2, LAS1L, LENG8, NOLC1, PARL, PISD, PUS1, SRRT, UNC50.

Table S2. shRNA sequences used in this study.

sh*MEN1*	CCGGGCTGCGATTCTACGACGGCATCTCGAGATGCCGTCGTAGAATCGCAGCTTTTTG
sh*KMT2A*	CCGGCGCCTAAAGCAGCTCTCATTTCTCGAGAAATGAGAGCTGCTTTAGGCGTTTTT
sh*MED12*	CCGGGCAGAGAAATTACGTTGTAATCTCGAGATTACAACGTAATTTCTCTGCTTTTT
sh*WAPL*	CCGGACGTACTGTAGGGCCAATAAACTCGAGTTTATTGGCCCTACAGTACGTTTTTTG
sh*GATA3*	CCGGCATCCAGACCAGAAACCGAAACTCGAGTTTCGGTTTCTGGTCTGGATGTTTTT

Table S3. RT-qPCR and Bio-mark primers used in this study.

Gene	F1 (5'- sequence -3')	R1 (5'- sequence -3')	R2 (5'- sequence -3')
MEN1	CCAGACAGTCAATGCCG	CAGGTCAATGGAAGGGTT	TGAACGCCACCTCCATC
KMT2A	AAATGAGAGTAATGATAGG AGAAGC	GTGGAGGTGGAGACGAG	TGGGGGGCACTTTGTAGA
MED12	TCAGTCAACAAGCACGC	AGGAGGATGGTCTGTAGG	GGGCACATAAGCAGGTC
WAPL	GTGACAAATCGGGGCTC	GGGTGGGTAGAAGGAAG AAA	TCAAAAATAGGAAGACAAT AGAAGGC
GATA3	CCACCACAACCACACTC	GGATTTGCTAGACATTTTT CGG	TGCCTTCCTTCTTCATAGT CAG
ALDOA	TCAAATCCAAGGGCGGT	CAATCTTCAGCACACAAC G	CTTGGGTGGTAGTCTCG
ALDOC	TTCACCTGGCACCAACT	GCCATCCTGTTCTGACTT CTA	GCTCCCTATCCTCCCATC
ENO1	GGAAAGATGCCACCAATG	CCCAGACCTGAAGAACT	CCAATAGCAGTCTTCAGC AG
PFKL	CGACTGGCTGTTCATCC	GAGATGGGCTTCCCGTT	CCCACGGCTCCGAGTCT
PFKP	GGAGAAGCACGAGGAGT	GCAGGTGTCGGTGATAGT	AGTGTTCAGGGCGGTGT
PGK1	TCGGGCTAAGCAGATTGT	CAAGTGGCAGTGTCTCC	GCTTTCACCACCTCATCC
TPI1	GAGCAGACAAAGGTCATC G	TGGACTTCAGCCATCCTC	CAGTCTTGCCAGTACCAA T
NDUFA7	ACTACTTTGCCTCCTTGG	GCCTGGGAAACATGGTG	AAAACTAGAAGTGATACCT GAGC
NDUFA11	AGTGAAACCCTGTCTCTAC TAT	GTGACATGATCTCGGCTC	TTCTCATGCCTCAGCCTC
NDUFA13	TACAGGCAGAAACCGACC	GTACAGCTCCCCGATCAA G	GAACACAGACTCCCCCAC
NDUFB7	CACACAGCAGGAGATGAT	GCAGTAGTCCCAGTCGT	CGCTTGCACTTGAGCAG
NDUFS7	GCACACTCACCAACAAGA	GATGTAGATGTCCACGGG	CATGGAGACCACGTAGCG
NDUFS8	ATCCCGAGATGGACATGA A	GTAGTTGATGGTGGCCG	GAACAGGTAGCTCAGGGT
NDUFV1	CTGTGGAGGAGGAGATGT	GGGGATCAGTGGGGTAG	AGTGGGGTAGACGAGCC
SDHA	CATTCCCACCAACTACAAG G	GACAACCAGGTCCAAGAG	TGTACCGAGGCACAGGC
SDHB	GAGAAGGCATCTGTGGC	GCATAGAAGTTGCTCAAA TCG	GTGTGGAAGAGGGTAGAT TT
SDHC	GTGGGTAGGGTTGAGGGA	ACATACAGTGGTTTGGGT G	CCAGAAATGACCAAGAGA AAG
SDHD	AACTATCACGATGTGGGC	CTTATCTGTTATTTCTTCC TTATTGTGAG	AAGCAGAGGCAAAGAGG
*CYC1**	CGGAGGTGGAGGTTCAA	CACGATGTAGCTGAGGTC	
*COX6C**	CGTATCAAGGACAGTAACT ACCA	ATCAGCCACACGAAACTT A	
*UQCR11**	GCTGACAGAAACTGCCTA	CCCAGATACATCACTCCC AA	
ACTB	CATGTACGTTGCTATCCAG GC	CTCCTTAATGTCACGCAC GAT	CTCCTTAATGTCACGCAC GAT

*RT-qPCR only, F1 and R1 was used. For *ACTB*, same primer set was used for outer and inner primer.
